# Enhancer transcription detected in the nascent transcriptomic landscape of bread wheat

**DOI:** 10.1186/s13059-022-02675-1

**Published:** 2022-04-30

**Authors:** Yilin Xie, Yan Chen, Zijuan Li, Jiafu Zhu, Min Liu, Yijing Zhang, Zhicheng Dong

**Affiliations:** 1 Guangdong Provincial Key Laboratory of Plant Adaptation and Molecular Design, Guangzhou Key Laboratory of Crop Gene Editing, Innovative Center of Molecular Genetics and Evolution, School of Life Sciences, Guangzhou University, Guangzhou, 510006 China; 2 National Key Laboratory of Plant Molecular Genetics, CAS Center for Excellence in Molecular Plant Sciences, Shanghai Institute of Plant Physiology and Ecology, Shanghai Institutes for Biological Sciences, Chinese Academy of Sciences, Shanghai, China; 3 University of the Chinese Academy of Sciences, Beijing, 100049 China; 4 State Key Laboratory of Genetic Engineering, Collaborative Innovation Center of Genetics and Development, Department of Biochemistry, Institute of Plant Biology, School of Life Sciences, Fudan University, Shanghai, 200438 China

**Keywords:** Bread wheat, Nascent RNA, Enhancer, eRNA, Subgenome-bias

## Abstract

The precise spatiotemporal gene expression is orchestrated by enhancers that lack general sequence features and thus are difficult to be computationally identified. By nascent RNA sequencing combined with epigenome profiling, we detect active transcription of enhancers from the complex bread wheat genome. We find that genes associated with transcriptional enhancers are expressed at significantly higher levels, and enhancer RNA is more precise and robust in predicting enhancer activity compared to chromatin features. We demonstrate that sub-genome-biased enhancer transcription could drive sub-genome-biased gene expression. This study highlights enhancer transcription as a hallmark in regulating gene expression in wheat.

**Supplementary Information:**

The online version contains supplementary material available at 10.1186/s13059-022-02675-1.

## Background

Widely cultivated wheat has an extremely large genome, which harbors abundant regulatory elements (REs) in noncoding regions [1, 2]. REs, including gene-proximal promoters and gene-distal enhancers, are key to the precise spatiotemporal regulation of gene expression [3, 4]. Enhancers affect the transcription of cognate genes independent of the relative distance, location or orientation [5–11]. Epigenomic studies revealed that REs often harbor particular chromatin features, including chromatin accessibility and certain histone marks, which have been widely used to characterize enhancers in both mammals and plants [1, 12–18]. Our labs and others recently reported that more than half of the regulatory hallmarks, including chromatin accessibility, histone modifications, and DNA methylation, are located in the intergenic regions of the wheat genome, suggesting the potential role of these epigenomic features in remote control of gene activity [1, 2]. However, an active chromatin environment does not necessarily mean an active enhancer [1, 19]. The transcription of enhancers has been widely detected by nascent RNA sequencing methods in animals [19–22], and enhancer RNAs (eRNAs) show more predictive power of enhancer activity than chromatin features [19]. Whether a plant enhancer can produce eRNA and its relevance to enhancer activity remain unknown.

## Results and discussion

**Fig. 1:**
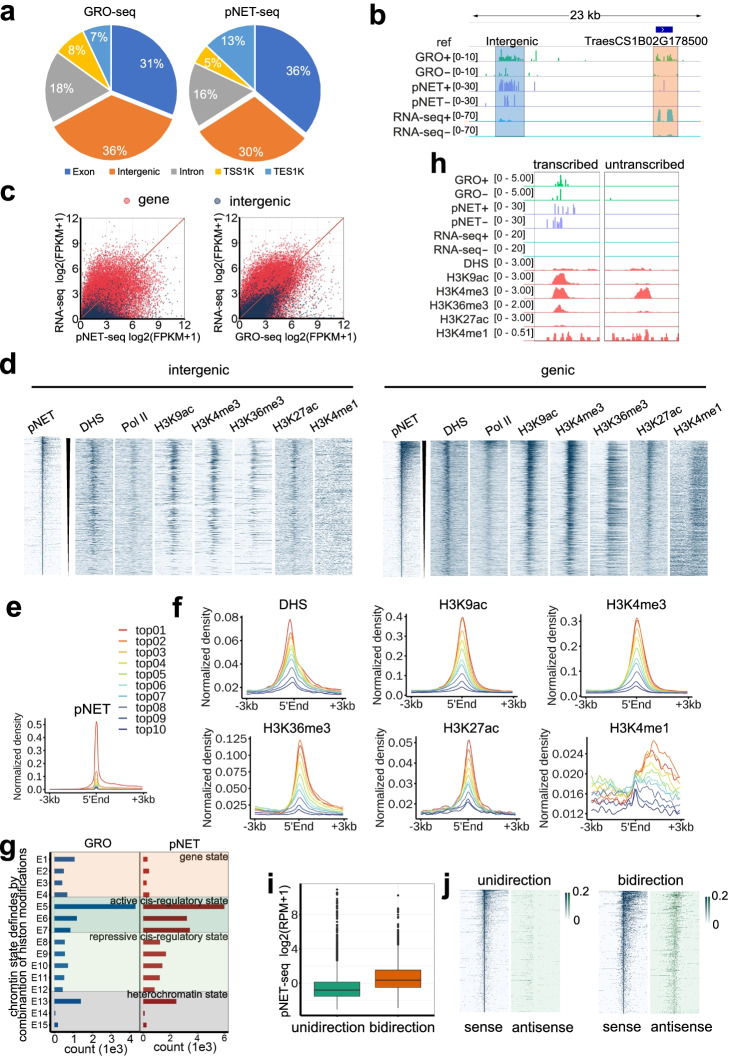
Unstable intergenic transcripts captured by GRO-seq and pNET-seq and eRNA characterization. **a** GRO-seq and pNET-seq reads aligned to different genomic regions. TSS1K indicates the 1 kb upstream region of the gene transcription start site. TES1K indicates the 1 kb downstream region of the transcription end site/polyadenylation site. **b** Browser shot of a protein-coding transcript and an intergenic transcript, the number of reads aligned to forward (+) and reverse (−) genomic strands are separately displayed. **c** Expression levels of nascent/steady-state transcripts from protein-coding and intergenic regions. **d** Read densities of pNET-seq, DHS and ChIP-seq of Pol II, H3K9ac, H3K4me3, H3K36me3, H3K27ac, and H3K4me1 around the intergenic and genic TCs (± 3 kb). All intergenic and genic TCs were ranked in a descending order of pNET-seq signals (±250 bp around the 5′ end), and chromatin features were plotted around the 5′ end of each intergenic TC. **e** pNET-seq signals around the intergenic TCs. Intergenic TCs were divided into ten equal parts based on the decreasing level of pNET-seq signals (± 250 bp). **f** Read densities of DHS and ChIP-seq of H3K9ac, H3K4me3, H3K36me3, H3K27ac, and H3K4me1 around each of the ten parts of intergenic TCs. **g** Intergenic TCs counts within different chromatin states defined in wheat genome [1]. **h** Chromatin signatures of transcribed and untranscribed enhancer examples. **i** Boxplots showing the expression levels of unidirectional and bidirectional enhancers determined by pNET-seq. **j** A heatmap displays the expression levels of unidirectional and bidirectional enhancers in primary (sense) and/or secondary (antisense) orientations (± 3 kb)

To answer these questions, we modified two nascent RNA sequencing methods for wheat seedlings: global nuclear run-on sequencing (GRO-seq) and plant native elongating transcript sequencing (pNET-seq) [23, 24]. Both methods capture nascent transcripts regardless of their stability, thus enabling the localization of the exact position, amount and orientation of transcriptionally engaged RNA polymerase II (Pol II) (Additional file 1: Figure S1). Overall, 13% of the bread wheat genome is revealed to be transcribed by nascent RNA-seq in contrast to 8% by conventional rRNA depleted strand specific RNA-seq (ssRNA-seq) in terms of uniquely mapped reads. Approximately 5–65 million (GRO-seq) and 26–40 million (pNET-seq) uniquely mapped reads were obtained from each biological replicate, with better reproducibility and more valid data for pNET-seq and rRNA removal GRO-seq than traditional GRO-seq (see Methods; Additional file 2: Supplementary Table 1). In the genome, genic regions are considered to be transcribed. Pol II-mediated transcription is slower in exons than in introns to facilitate splicing recognition. Therefore, it is likely that more nascent RNAs are detected in exons than in introns [25]. As expected, one of the highest fractions of the aligned reads was derived from exons (Fig. 1a). Notably, the other highest proportion is transcribed from intergenic regions. In contrast, the Arabidopsis genome is less than 1% of the wheat genome, and only 18% is intergenic. Transcription was detected in 19% of Arabidopsis intergenic regions, accounting for 1% of nascent RNAs detected by GRO-seq (Additional file 1: Figure S2a). Compared with protein-coding transcripts, intergenic transcripts in the wheat genome are more likely to be involved in quick turnover events and therefore cannot be comprehensively detected by ssRNA-seq. Figure 1b shows a specific example of adjacent intergenic and protein-coding transcription within a 23 kb region, where strong GRO-seq and pNET-seq signals are found at both regions, while ssRNA-seq signals are confined to the protein-coding region. Moreover, the genome-wide read coverage of GRO-seq or pNET-seq was significantly higher than that of ssRNA-seq, and the gap widened with increasing sequencing depth (Additional file 1: Figure S2b). Not surprisingly, this gap was also observed in other species including human [26, 27] and maize [28, 29], but not in Arabidopsis [23], which has a compact genome and much fewer intergenic regions.

Consistent with previous studies, apparent transcriptional pausing was observed in close proximity to active transcription start sites (TSSs) of annotated genes [23, 24] (Additional file 1: Figure S1, S2c). In total, we obtained 1,044,770 (GRO-seq) or 88,830 (pNET-seq) TSS clusters (TCs) through TSScall [20], of which a significant number were found to be within 1 kb upstream or downstream of the annotated genes (see Methods; Additional file 1: Figure S1, S2c; Additional file 3: Supplementary Table 2). As shown in Figure S3, the gene-proximal TCs identified above are indeed enriched around annotated TSSs, suggesting that TSScall is a robust approach for global TSS identification. Among the detected TCs, 45,872 (GRO-seq, 43.8%) and 33,355 (pNET-seq, 37.5%) were localized to intergenic regions, and intergenic TCs displayed higher nascent RNA levels than mature RNA levels, whereas no such bias was observed for protein coding transcripts (Fig. 1c). The overall correlations of transcription clusters between GRO-seq and pNET-seq were 0.57 and 0.60 at genic and intergenic regions, respectively (Additional file 1: Figure S4). Collectively, these results demonstrate that intergenic transcripts are indeed much less stable than gene transcripts and that the GRO-seq and pNET-seq combination is more sensitive in detecting intergenic transcripts than mature RNA sequencing.

We next characterized the epigenomic features surrounding intergenic and genic transcribed regions. Overall, both intergenic and genic transcription are strongly associated with open chromatin regions, RNA Pol II engagement and transcriptionally active histone marks including H3K9ac, H3K4me3, H3K36me3, and H3K27ac (Fig. 1d; Additional file 1: Figure S5). To investigate the relationship between transcription and chromatin features, we divided the intergenic TCs into ten groups according to their transcriptional activity determined by pNET-seq and plotted the epigenetic profiles surrounding the 5′ end of intergenic TCs (Fig. 1e, f). We observed that intergenic transcription was positively correlated with chromatin accessibility and the active histone marks H3K9ac and H3K4me3 as mentioned above (Fig. 1f). Within the genic region, the peaks of H3K9ac, H3K4me3, H3K36me3, and H3K27ac were biased to the transcriptional direction, while the peaks in the intergenic regions were symmetric (Fig. 1f; Additional file 1: Figure S6). These patterns suggest that the expression directions of genic and intergenic regions are different. Consistent with previous plant epigenome studies [1, 30], more H3K4me1 was detected in gene bodies than in intergenic regions (Fig. 1d; Additional file 1: Figure S5). However, there was a weak correlation between nascent RNA abundance and H3K4me1 levels in intergenic regions, whereas there was no clear relationship between H3K4me1 and nascent RNA levels in genes (Fig. 1f; Additional file 1: Figure S6). Analyses based on GRO-seq data led to the same results (Additional file 1: Figure S7). Together, transcribed intergenic regions were essentially marked by active chromatin states.

Given that intergenic transcribed regions in wheat hold a variety of active regulatory marks, we speculated that a fraction of intergenic TCs represents eRNAs. As indicated in Figure S8a, an intergenic transcribed region displayed high levels of enhancer hallmarks [1], including open chromatin, H3K9ac, H3K4me3, H3K36me3, and H3K27ac. Previously, we categorized the wheat genome into 15 chromatin states based on combinatorial histone modification marks using a multivariate hidden Markov model (HMM) which has been applied in detection of REs related to individual marks. Chromatin states 5–7 were marked by high DNA accessibility, histone acetylation, CpG islands, and relatively conserved sequences. Therefore, we defined the chromatin state 5–7 regions, which are 3 kb distant from the nearest gene, as enhancer-like elements. In a tobacco transient system, approximately half of the putative enhancer-like elements increase the expression of the reporter gene [1]. A total of 25% (11,484 out of 45,872, GRO-seq) and 38% (12,687 out of 33,355, pNET-seq) of intergenic TCs were from states 5–7 (Fig. 1g). In fact, the proportions of intergenic enhancer-like elements being transcribed in states 5–7 were significantly higher than those in other states (Additional file 1: Figure S9). When compared to those that were untranscribed, the transcribed enhancer-like elements showed higher accessibility, more active histone marks, fewer repressive histone marks, and lower levels of CpG methylation (Additional file 1: Figure S8b, c; Fig. 1 h). No observable difference was uncovered between these two types of enhancers with respect to H3K4me1. Although the high H3K4me1 to H3K4me3 ratio is supposed to be a typical enhancer feature in mammals [1, 31, 32], emerging evidence in both *Drosophila* and mammals indicates that highly active enhancers are generally marked by H3K4me3 [20, 33–36], which is consistent with our results that transcribed enhancers are significantly enriched with H3K4me3 but not H3K4me1 (Additional file 1: Figure S8c). We next wondered whether there are sequence signatures within enhancers and found that WRKY, GeBP, zfGRF, and MYB-related transcription factor binding motifs were specifically overrepresented in enhancers rather than promoters (Additional file 1: Figure S10a). Moreover, these motifs are significantly enriched in transcribed enhancers compared with untranscribed enhancers (Additional file 1: Figure S10b).

Bidirectional transcription at enhancers and promoters has been widely discovered in mammals [37]. However, from Arabidopsis [23] and the wheat genome, no significant bidirectional promoter was observed (Additional file 1: Figure S2c). We then ask whether this is the case for plant enhancers. A total of 4407/2144 (pNET-seq/GRO-seq) bidirectional transcribed enhancers (BEs) and 8280/9340 (pNET-seq/GRO-seq) unidirectional transcribed enhancers (UEs) were revealed. Explicitly, here, for enhancers, the strand more actively transcribed is defined as the primary strand and the other strand is secondary. Corresponding transcripts are considered to be in primary or in secondary direction. From the pNET-seq assay, the transcriptional levels of BEs were found to be significantly higher than those of UEs in both the primary and secondary directions (*p* < 2.2E−16, Welch’s two-sample *t*-test) (Fig. 1i), as evidenced by the stronger pNET-seq signals around the 5′ end of BEs (Fig. 1j). However, the expression levels of BEs were lower than UEs according to GRO-seq (Additional file 1: Figure S11a, b).

**Fig. 2:**
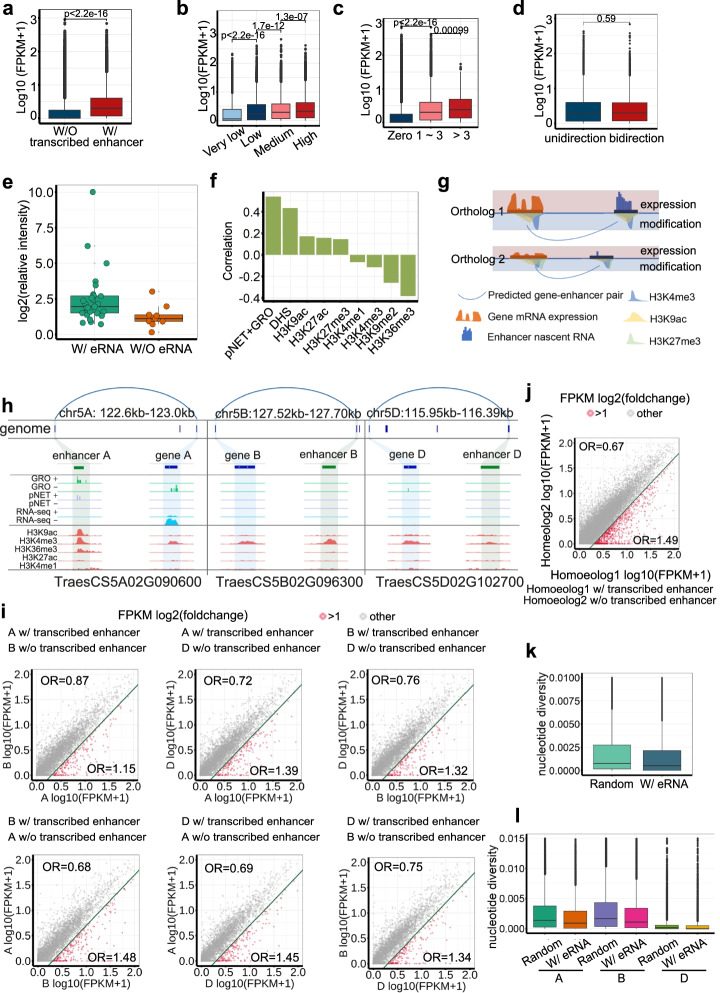
A putative role of enhancer transcription in regulating subgenome-divergent gene expression and conservation of eRNA regions. **a**–**d** Boxplots showing transcript levels of target genes. **a** Genes associated with transcribed enhancer(s) display higher expression levels. **b** The enhancer transcriptional level positively correlates with its target gene expression. **c** The number of associated transcribed enhancers positively correlates with its target gene expression. **d** Genes targeted by unidirectional or bidirectional enhancers show no significant differences in expression levels. **e** The enhancer activity (relative intensity) determined in wheat protoplasts of transcribed and untranscribed enhancer candidates. **f** Correlations between the enhancer activity and the nascent RNA-seq, DHS, and ChIP-seq read density in predicted enhancer regions. **g** Schematic illustration of the biased expression homeolog genes targeted by enhancers with different transcription levels. Histone modification correlations and physical interactions between enhancers and putative target genes were counted. **h** Snapshot of a triad (*PR10*) with subgenome-biased expression and eRNAs. **i** Subgenome biased transcribed enhancers are enriched with unbalanced expressed homoeologs between each two subgenome pairs (from pNET-seq data). Each dot represents a gene pair in which one homeolog is associated with at least one transcribed enhancer in the same chromosome, while the other homeolog is not. The *X*-axis and *Y*-axis coordinates represent the expression levels of the two homeolog genes respectively. Red dots represent gene pairs in which the expression level of a homeolog gene associated with transcribed enhancer(s) was significantly higher than that of the other one not associated with transcribed enhancer; while the grey dots represent a homeolog gene associated with transcribed enhancer(s) was equally or lower expressed than the other one. “A w/ transcribed enhancer, B w/o transcribed enhancer” means homeolog A is associated with transcribed enhancer(s), but homeolog B is not. The odds ratio (OR) is a ratio of two sets of odds to measure the degree to which homeolog gene expression is correlated with its association with enhancer(s). See detailed analyses and interpretation in Additional file 1: Figure S15. **j** Merge all homoeolog pairs in **i** showed that the dominantly expressed homoeologs essentially associated with transcribed enhancers. **k**, **l** Nucleotide diversity distribution of enhancers with eRNA and random intergenic regions across the genome (**k**) and within A, B, and D subgenomes (**l**)

To further elucidate the biological significance of enhancer transcription, we linked these transcribed enhancers to putative gene targets. Herein, we employed the information from both epigenetic correlation and direct chromatin interaction between transcribed enhancers and gene promoters to define the targets of enhancers (see Methods; Additional file 1: Figure S1), resulting in 9925/9299 (pNET-seq/GRO-seq) transcribed enhancers connected with at least one target and 26,712/27,092 (pNET-seq/GRO-seq) genes targeted by at least one transcribed enhancer (Additional file 4: Table S3 and Additional file 5: Table S4). As indicated in Fig. 2a and Fig. S11c, genes associated with transcribed enhancer(s) displayed significantly higher expression levels than those without a transcribed enhancer. Further quantitative analysis revealed a positive correlation between enhancer and target transcription (Fig. 2b, Additional file 1: Figure S11d, S12). It is worth noting that none of the epigenetic markers except H3K9ac enriched in enhancers showed a quantitative association with target gene expression (Additional file 1: Figure S12). Moreover, the greater the number of associated transcribed enhancers, the higher the expression level of the target genes (Fig. 2c, Additional file 1: Figure S11e). To determine whether the direction of enhancer transcription would affect target gene expression, we calculated the expression levels of genes associated with unidirectional or bidirectional enhancers. The results showed that the target gene expression level is independent of the transcriptional direction of the associated enhancers (Fig. 2d). Likewise, enhancers in wheat regulate target gene expression regardless of the distance, position, and orientation (Additional file 1: Figure S13).

We then asked whether the enhancer transcription level was related to enhancer activity. We tested the “enhancer activity” of 36 putative intergenic enhancers with or without eRNA, which was determined using *gfp* or dual *luciferases* as reporters in wheat protoplasts (see Methods; Additional file 1: Figure S14, S1). The putative enhancer region was fused with a 35S minimal promoter (mini 35S pro) controlling the transcription of reporter genes, and the “enhancer activity” was defined as its effect on the reporter gene expression in comparison to the blank and negative controls, measured qualitatively by fluorescence of GFP or quantitatively by luciferase activity. The transcribed candidate enhancers were more active than untranscribed ones in wheat protoplasts (Fig. 2e; Additional file 1: Figure S15a). Furthermore, the positive candidate enhancers validated in the wheat protoplasts had higher transcriptional activity in wheat seedlings (Additional file 1: Figure S15b). Pearson correlation coefficients were calculated between the relative luminescence intensities (enhancer activity) and the read densities of nascent RNA (pNET-seq and GRO-seq) and different chromatin marks. Notably, enhancer activity was the most strongly correlated with enhancer transcription (GRO-seq and pNET-seq, *r* = 0.55), followed by chromatin accessibility (DHS, *r* = 0.43) and active histone modification (H3K9ac, *r* = 0.17; and H3K27ac, *r* = 0.16), which indicated that eRNA is more robust in predicting enhancer activity than canonical chromatin features (Fig. 2f).

For hexaploid, subgenome-divergent transcription was proposed as a major factor contributing to the superior adaptability and agronomic traits of bread wheat [38, 39]. Approximately 30% of wheat homoeolog triads are unevenly expressed, with one homoeolog being dominant or recessive relative to the other two [40]. Previous studies have demonstrated that subgenome-biased expression among homoeolog triads is closely associated with both epigenetic divergence and variation in transposable elements within promoter regions across subgenomes (Fig. 2g) [1, 40–42]. Here, we found a homoeolog triad of the *Pathogenesis-Related 10* (*PR10*) gene, which is differentially expressed in various tissues [43] and is associated with differentially expressed enhancers. *PR10* from subgenomes A was targeted by a transcribed enhancer and displayed higher expression levels than the other two homoeologs from subgenomes B and D, which were associated with untranscribed enhancers (Fig. 2h). To verify whether subgenome-divergent gene expression is related to subgenome-biased enhancer transcription in addition to epigenetic modifications (Fig. 2g), we focused on 67,108 pairs of homoeolog genes that have 1:1 correspondence between any two of the three subgenomes, using the odds ratio (OR) to measure how strongly “one homeolog gene being expressed higher than the other gene” is related to “one is associated with transcribed enhancer(s) but the other is not” (Additional file 1: Figure S16). We found that subgenome-biased gene expression was significantly enriched with subgenome-biased enhancer transcription (Fig. 2i, Additional file 1: Figure S17a). Specifically, dominantly expressed homoeologs were essentially associated with transcribed enhancers, at an OR of 1.49 (pNET-seq) or 1.64 (GRO-seq) (Fig. 2j, Additional file 1: Figure S17b).

Finally, we addressed whether transcribed enhancer regions were conserved in the natural wheat population. The nucleotide diversity was lower in the enhancer regions with eRNA than in the randomly chosen intergenic regions both across the genome and within each subgenome (Fig. 2k, l, Additional file 1: Figure S11f, g), which suggested that eRNA regions were selected for in the genetic improvement of wheat. We further examined the wheat eRNA homoeologous regions in the grass family; however, eRNA regions were no more conserved than the randomly picked intergenic regions (Additional file 1: Figure S18, S19). Taken together, eRNA coding regions are conserved among different wheat cultivars but not different grass species.

## Conclusions

The key insights into enhancer transcription, a defining nature for active enhancers, have thus far been provided by studies in *Drosophila* and mammals. However, the first nascent RNA study in plants demonstrated that few transcripts are produced from intergenic enhancers in Arabidopsis [24]. This is probably due to the relatively compact genome that contains fewer distal regulatory elements. Here, we surveyed the 16 Gb bread wheat genome comprising large intergenic regions and a wide variety of REs, including enhancers. By nascent RNA profiling, we detected actively transcribed enhancers in wheat. The overall chromatin signature of transcribed enhancers is quite similar to that of actively transcribed genes, marked with open chromatin, H3K9ac, H3K4me3, H3K36me3, and H3K27ac. However, bidirectional transcription was often observed at enhancers rather than at genic regions. The transcription levels of enhancers effectively predict the transcriptional status of their target genes in wheat. Furthermore, the subgenome-biased target gene expression was significantly enriched with subgenome-biased enhancer transcription, indicating that enhancer transcription should be one of the reasons for the asymmetric expression patterns between subgenomes and could be a reliable indicator of enhancer activity. As further genetic and biochemical evidences is required to dissect their functional mechanisms in plants, enhancers/eRNAs could be breeding targets either by traditional crossing or advanced genome editing in future crop improvement.

## Methods

### Plant materials and growth conditions

The bread wheat (*Triticum aestivum*) cultivar “Chinese Spring” was grown under long-day conditions. Leaf tissue of 12-day-old seedlings was collected by flash freezing with liquid nitrogen followed by nuclei isolation.

### GRO-seq experiment

Nuclei were isolated by the Percoll gradient procedure as previously described except that the concentration of Triton X-100 was reduced to 0.5% [23]. In vitro run-on and nascent RNA purification were performed as previously reported [23]. The obtained nascent RNA was treated with T4 PNK (NEB), followed by TRIzol purification and subject to small RNA library construction using NEXTflex™ Small RNA-Seq Kit v3 (PerkinElmer) according to the manufacturer’s manual. We performed two rounds of GRO-seq. For the first time (rep1 and rep2), the ratio of unique mapping reads (we used for further transcription analyses) to total raw reads was 1% (Additional file 2: Supplementary Table 1). For the second time (v2_rep1, v2_rep2, and v2_rep3), to obtain a deeper sequencing coverage at a reasonable sequencing cost, we added an rRNA removal step after nuclear RNA isolation and before the affinity purification of nascent RNA using riboPOOL kit (siTOOLs BIOTECH). Then, the unique mapping ratio was increased to 16% (Additional file 2: Supplementary Table 1). The cDNA libraries were sequenced on an Illumina NovaSeq platform by Shanghai Hanyu Biotech. The two rounds of GRO-seq data were then merged for further analyses.

### pNET-seq experiment

Nuclei were isolated by a lysis-and-wash procedure as previously described except that the concentration of Triton X-100 was reduced to 0.5% [23]. The RNA Pol II-associated RNA was immunoprecipitated using Pol II antibody (Abcam, ab817), followed by small RNA cDNA library construction [23]. Ab817 was shown to recognize both unphosphorylated and phosphorylated wheat RNA Polymerase II by immunoblotting with total protein from control and flavopiridol-treated seedling tissue (Additional file 1: Figure S20). The size selection of nascent RNA was omitted. The cDNA libraries were sequenced on an Illumina NovaSeq platform by Shanghai Hanyu Biotech.

### GRO-seq data mapping and processing

Sequencing reads were cleaned with Trim Galore (version 0.6.4) and cutadapt (version 1.16) programs [44] to remove sequencing adapters, low quality bases (< 20), short reads and the 4 bases in the 5′ end and 3′ end of R1 and R2 reads, which were randomly introduced. Clean reads were aligned to the International Wheat Genome Sequencing Consortium (IWGSC) reference sequence (version 1.0) with the bowtie2 (version 2.3.5.1) program [45], and only the reads with one best unique alignment were retained. We also used the SortMeRNA (version 2.1b) program [46] to remove the reads originating from chloroplasts, mitochondria, and rRNA. The 5′ coordinate of R1 reads was considered the position of the engaged polymerase for future analyses. Each site density of GRO-seq was then calculated in plus and minus strands and normalized by the count of unique mapped reads. The correlations of the biological replicates were calculated by the read density in 500-bp bins, and the alignments were then combined to obtain a higher depth for subsequent analysis.

### pNET-seq data mapping and processing

Sequencing reads were cleaned and mapped as described above except the 5′ coordinate of R2 reads with the directionality indicated by R1 reads was considered the position of the engaged polymerase. The pNET-seq signal at each site and the correlations of the 4 biological replicates were calculated as described above. The alignments of 4 biological replicates were combined for subsequent analysis.

### Intergenic transcript annotation

TSScall [20] with the default parameter was applied to define the GRO-seq and pNET-seq transcripts. Although this program was designed to identify TSSs from Start-seq data, visual inspection found that it performs well in calling short transcript start sites of GRO-seq and pNET-seq. For longer transcripts, it may call multiple peaks along with one transcript. Therefore, the nearby TSSs within 3 kb of one another were merged into TSS clusters (TCs). The 5′ end of the TC were selected as the TSS of this transcript. To avoid random sites and noise obtained by the experimental technique, only the TSSs found in all replicates were retained.

With the aim of accurately achieving the intergenic transcript, we removed the TCs that overlapped with the annotated high confidence and low confidence genes (IWGSC v1.1). To avoid false-positives due to incomplete annotations, we also removed the TCs that overlapped with the upstream and downstream 1 kb of annotated genes. In addition, we applied StringTie [47] and CPC2 [48] to assemble and predict the potential protein coding transcripts using multitissue ssRNA-seq alignments. TCs with overlap with these transcripts were also removed.

### Comparison of genomic coverage in bread wheat and other species

Human data were downloaded from NCBI (https://www.ncbi.nlm.nih.gov/geo/query/acc.cgi?acc=GSM2400210 for RNA-seq and https://www.ncbi.nlm.nih.gov/geo/query/acc.cgi?acc=GSM340901 for GRO-seq). Maize data were downloaded from NCBI (https://www.ncbi.nlm.nih.gov/geo/query/acc.cgi?acc= GSM2041246 for RNA-seq and https://www.ncbi.nlm.nih.gov/geo/query/acc.cgi?acc=GSM1309038 for GRO-seq). Arabidopsis data were download from NCBI (https://www.ncbi.nlm.nih.gov/geo/query/acc.cgi?acc=GSM2974957 for RNA-seq and https://www.ncbi.nlm.nih.gov/geo/query/acc.cgi?acc=GSM2974947 for GRO-seq).

For each dataset, we randomly selected 0.1–80 million alignments from the bam files, calculated the coverage in the 10-kb bins of the corresponding genome and set the cutoff to 5 to determine whether the bin was covered.

### RNA-seq data processing

RNA-seq data used for comparison with nascent RNA-seq data were downloaded from NCBI (https://www.ncbi.nlm.nih.gov/geo/query/acc.cgi?acc=GSM3449733). The RNA-seq dataset used to assemble transcripts with coding potential was downloaded from NCBI (https://www.ncbi.nlm.nih.gov/geo/query/acc.cgi?acc=GSE139019). The sequencing reads were trimmed with Trim Galore (version 0.6.4), and clean reads were mapped to the IWGSC reference sequence (version 1.0) with the HISAT2 program (version 2.1.0) [49]. The featureCount program of the Subread package (version 1.5.3) [50] was used to determine the RNA-seq read density of the high-confidence genes in the IWGSC RefSeq genome assembly (version 1.1). The RNA-seq read density of each gene was normalized based on the exon length in the gene and the sequencing depth [i.e., fragments per kilobase of exon model per million mapped reads (FPKM)]. Sixteen RNA-seq samples were used to assemble the potential transcripts using the StringTie program (2.1.4) [47]. The coding potential was predicted by the CPC2 program [48].

### Bisulfite-seq, ChIP-seq, and DNase-seq data processing

Bisulfite-seq, ChIP-seq, and DNase-seq data of Chinese spring seedling tissue were downloaded from NCBI (https://www.ncbi.nlm.nih.gov/geo/query/acc.cgi?acc=GSE121903). The reads were cleaned and mapped as described previously [1]. To compare the differences in the methylation modification levels in different intervals of the genome, the average methylation ratios in the 500-bp bins of the whole genome were calculated.

### Motif enrichment analysis

The motifs were analyzed with “findMotifs.pl” implemented in Homer software [51]. Taking random genomic regions as the background, the 500-bp region flanking the promoters or the predicted enhancers was chosen as the primary sequence input, and then, we calculated the enrichment fold change of each known motif in plants.

Taking promoter sequence as the background and the predicted enhancer sequence as the input and vice versa, we found motifs that were differentially enriched in the promoter and enhancer. Then, with random genomic regions as background, we calculated the enrichment fold change of these motifs in transcribed and untranscribed enhancers separately.

### Assignment of transcribed enhancers to target genes

We searched the transcribed enhancers and target gene pairs according to the distance, the correlation of histone modification, and the 3D interaction intensity between them. First, only genes within 500 kb of the enhancer are considered. The recently published modification peaks were used [52], and we chose the peaks that overlapped with the enhancer and promoter pairs to calculate the correlation. The cutoff was set to 0.7. Hi-C data were used to count the interaction intensity between the candidate pairs [53]. The filtered result must be connected by at least one Hi-C read pair. If no eligible target gene was found within 500 kb, then the search range was expanded to 2 Mb.

### Verification of enhancer activity by a luciferase reporter assay in wheat protoplasts

Sequences of the predicted intergenic enhancers were amplified from Chinese Spring genomic DNA (primers are listed in Additional file 6: Table S5) and fused with a minimal 35S promoter, which drives the transcription of a *Fluc* (*firefly luciferase*) or *gfp* (*green fluorescent protein*) gene [1] (Additional file 1: Figure S14). Wheat protoplast preparation and transformation were performed according to a previously reported protocol [54]. Briefly, wheat leaves were cut into strips with a razor blade and then incubated with cellulase and macerozyme to release protoplasts. A total of 15 μg plasmids were transformed into 1 × 10^5^ protoplasts using the PEG-mediated transfection method. After 24 h of incubation under dark conditions at room temperature, protoplasts were collected for visualization of fluorescence or luciferase assays. The protoplasts transformed with *gfp* reporter constructs were observed with a confocal microscope (Nikon C2 plus) (Additional file 1: Figure S15a). For the dual luciferase reporter system, *Rluc* (*Renilla luciferase*) driven by a *UBQ10* promoter was used to monitor transfection efficiency in the same vector (Additional file 1: Figure S14). The luciferase assay was completed using the Dual-Luciferase Reporter Assay System (Promega) according to the manufacturer’s instructions on a BioTek Synergy 2 microplate reader. The relative expression level of each construct was defined as the ratio of Fluc to Rluc activity. To calculate the enhancer activity (relative intensity), the relative expression level of each enhancer candidate construct was normalized to that of the blank control, in which *Fluc* was only driven by the minimal 35S promoter. An intergenic region where there were no DHS, pNET-seq, and GRO-seq signals was used as a negative control. The average of three independent biological replicates was recorded for each construct. The enhancer activity (relative intensity) of the negative control was 1.6; thus, we set 2.0 as the cutoff of the positive enhancer. The activity for each enhancer candidate is listed in Additional file 7: Table S6. For correlation coefficient calculation between enhancer activity and eRNA/histone modification/DHS levels, extreme out group points were omitted.

### Nucleotide diversity calculation

Twenty whole genome resequencing data were downloaded from NCBI (https://www.ncbi.nlm.nih.gov/bioproject/PRJNA476679) [55]. The reads were cleaned and mapped as described previously [1]. The low-quality and duplicate reads were removed. SNP/indel detection was performed using the GATK HaplotypeCaller GenotypeGVCFs (version 4.1.9.0) set for diploids with default filtering settings [56]. Then, SNPs were further excluded by Plink (v1.90b6.18) [57] with the parameter “-geno 0.05 -mind 0.05 -hwe 0.0001 -maf 0.05”. The nucleotide diversity was calculated using vcftools [58] in 500 bp windows across the genome.

## Supplementary Information

**Additional file 1: Figures S1**. A diagram of the experimental approach. **Figures S2-S4**. GRO-seq and pNET-seq data quality. **Figures S5-S10**. Features of genic and intergenic transcription loci. **Figures S11-S13**. Characterization of eRNAs. **Figures S14-S15**. Validation of enhancer activity in wheat protoplasts. **Figures S16-S17**. eRNA and sub-genome-biased gene expression. **Figures S18-S19**. Conservation of eRNA regions [67]. **Figure S20**. Validation of anti-Pol II antibodies. **Figure S21**. Uncropped images for Figure S20.

**Additional file 2: Supplementary Table S1**. Statistics of nascent RNA sequencing data quality.

**Additional file 3: Supplementary Table S2**. TSS and TC counts identified by TSScall.

**Additional file 4: Supplementary Table S3**. Transcribed enhancer counts linked to homeolog pairs (pNET).

**Additional file 5: Supplementary Table S4**. Transcribed enhancer counts linked to homeolog pairs (GRO).

**Additional file 6: Supplementary Table S5**. Genomic positions and primers used for the reporter assay.

**Additional file 7: Supplementary Table S6**. Location, eRNA level, and enhancer activity of 36 putative enhancers tested in wheat protoplasts.

**Additional file 8.** Review history.

## Data Availability

The GRO-seq and pNET-seq data of bread wheat in this study are available in the Gene Expression Omnibus (GEO) database under accession number GSE178276 [59]. RNA-seq, BS-seq, ChIP-seq, and DNase-seq data of bread wheat were published previously [60, 61]. For comparison of genomic coverage, both RNA-seq and nascent RNA-seq data of bread wheat were analyzed in parallel with the data of other species including human [62, 63], maize [64, 65], and *Arabidopsis* [66].
