# Supplementary material for: Enhancer transcription detected in the nascent transcriptomic landscape of bread wheat
Source: Genome Biol. 2022 Apr 30;23:109. doi: 10.1186/s13059-022-02675-1 (PMC9063354; doi:10.1186/s13059-022-02675-1)
Supplement: Supplementary file 1 — Additional file 1: Figures S1. A diagram of the experimental approach. Figures S2-S4. GRO-seq and pNET-seq data quality. Figures S5-S10. Features of genic and intergenic transcription loci. Figures S11-S13. Characterization of eRNAs. Figures S14-S15. Validation of enhancer activity in wheat protoplasts. Figures S16-S17. eRNA and sub-genome-biased gene expression. Figures S18-S19. Conservation of eRNA regions [67]. Figure S20. Validation of anti-Pol II antibodies. Figure S21. Uncropped images for Figure S20. [file 13059_2022_2675_MOESM1_ESM.pdf]

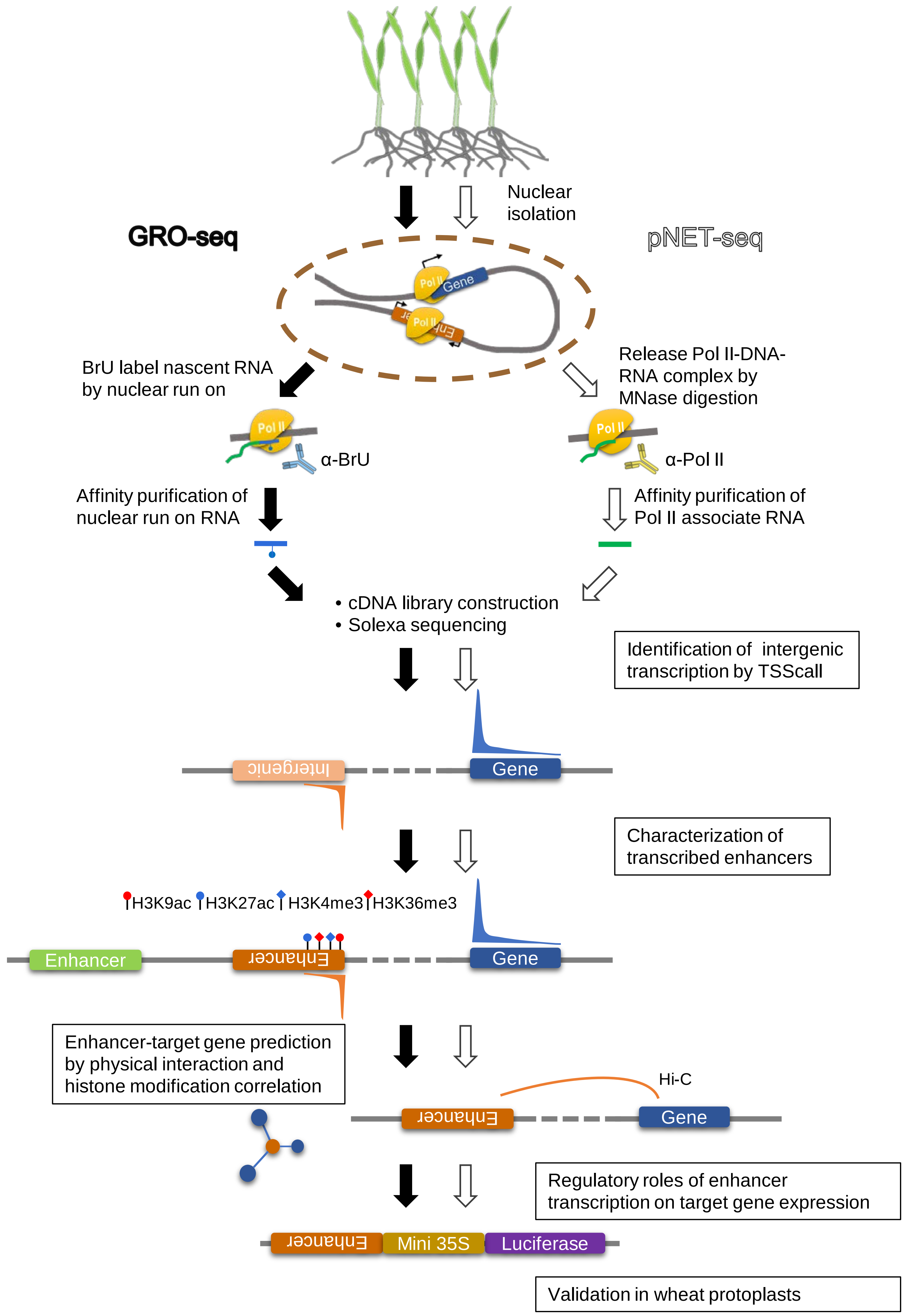

Nuclear isolation
GRO-seq
pNET-seq
Pol II
Gene
Pol II
Enh
BrU label nascent RNA by nuclear run on
Release Pol II-DNA-RNA complex by MNase digestion
Pol II
α-BrU
Pol II
α-Pol II
Affinity purification of nuclear run on RNA
Affinity purification of Pol II associate RNA
• cDNA library construction
• Solexa sequencing
Identification of intergenic transcription by TSScall
Intergenic
Gene
Characterization of transcribed enhancers
H3K9ac H3K27ac H3K4me3 H3K36me3
Enhancer
Enhancer
Gene
Enhancer-target gene prediction by physical interaction and histone modification correlation
Hi-C
Enhancer
Gene
Regulatory roles of enhancer transcription on target gene expression
Enhancer
Mini 35S
Luciferase
Validation in wheat protoplasts

Figure S1. A diagram of the experimental approach.
GRO-seq or pNET-seq strategies were adopted for identifying nascent transcripts. Detailed procedures for GRO-seq and pNET-seq are as described in Methods. Nuclei were isolated from wheat seedling. For GRO-seq, nascent RNAs were labeled with BrU by nuclear run on and affinity purified with anti-BrU antibody. For pNET-seq, nascent RNAs were obtained by immunopreciptating Pol II associate RNA. Nascent RNAs were then converted to cDNA libraries and sequenced. Reads from high throughput sequencing were aligned to the wheat reference genome. The 5' end of GRO-seq reads and 3' end of pNET-seq reads display strong peaks in close proximity to active TSSs, which are identified by TSScall as proximal TSSs. Intergenic transcription was therefore defined and superimposed to the distal enhancers previous defined by chromatin states. Chromatin features of transcribed enhancers were characterized. Enhancer-gene regulatory links were predicted by the epigenetic correlation and long distance physical interaction between promoters and transcribed enhancers. The regulatory roles of enhancer transcription on target gene expression were analyzed and confirmed by transient reporter assay in wheat protoplast.

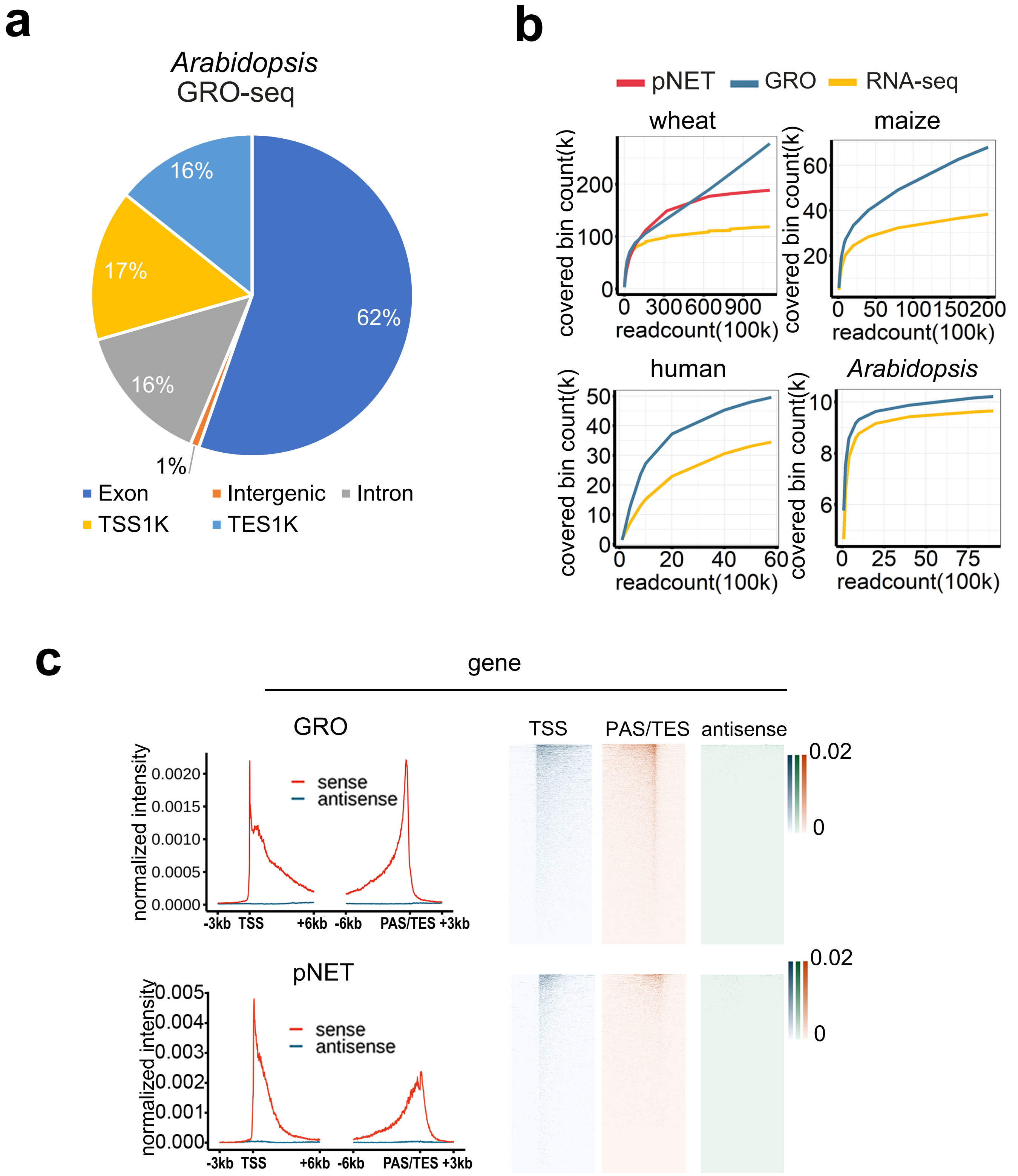

Figure S2. Detect wheat transcription by nascent RNA sequencing methods.
a, GRO-seq reads aligned to different Arabidopsis genomic regions.
TSS1K indicates the 1 kb upstream region of the gene transcription start site.
TES1K indicates the 1 kb downstream region of the gene transcription end site/polyadenylation site.
b, Genomic coverage of ssRNA-seq, GRO-seq and pNET-seq with different read counts in wheat, human, maize and Arabidopsis.
c, GRO-seq and pNET-seq signals along annotated genic regions.

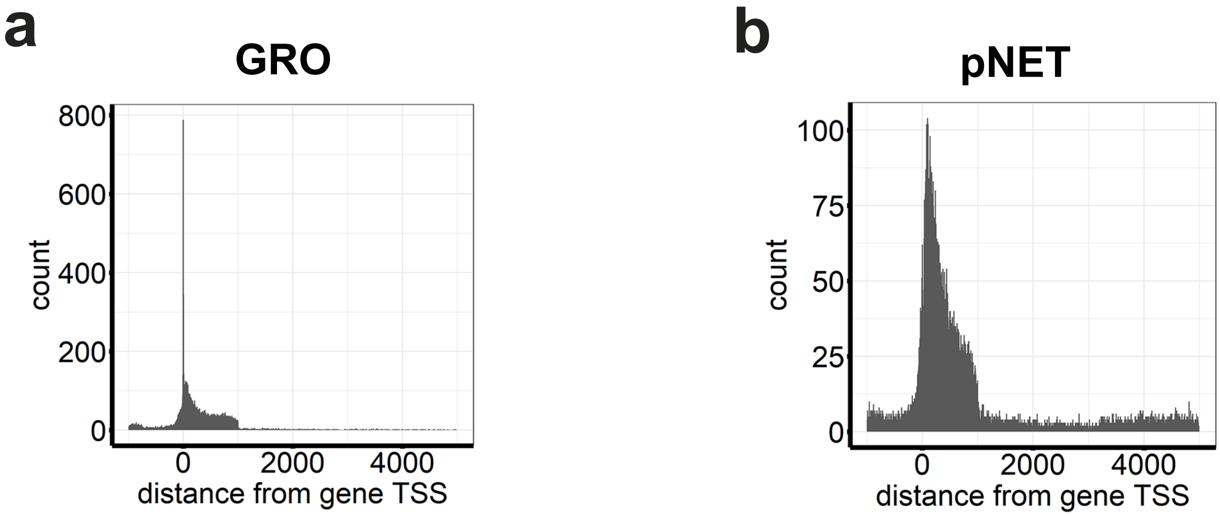

Figure S3. Distribution of gene-proximal TSS clusters (TCs) defined by GRO-seq (a) or pNET-seq (b) around annotated TSSs.

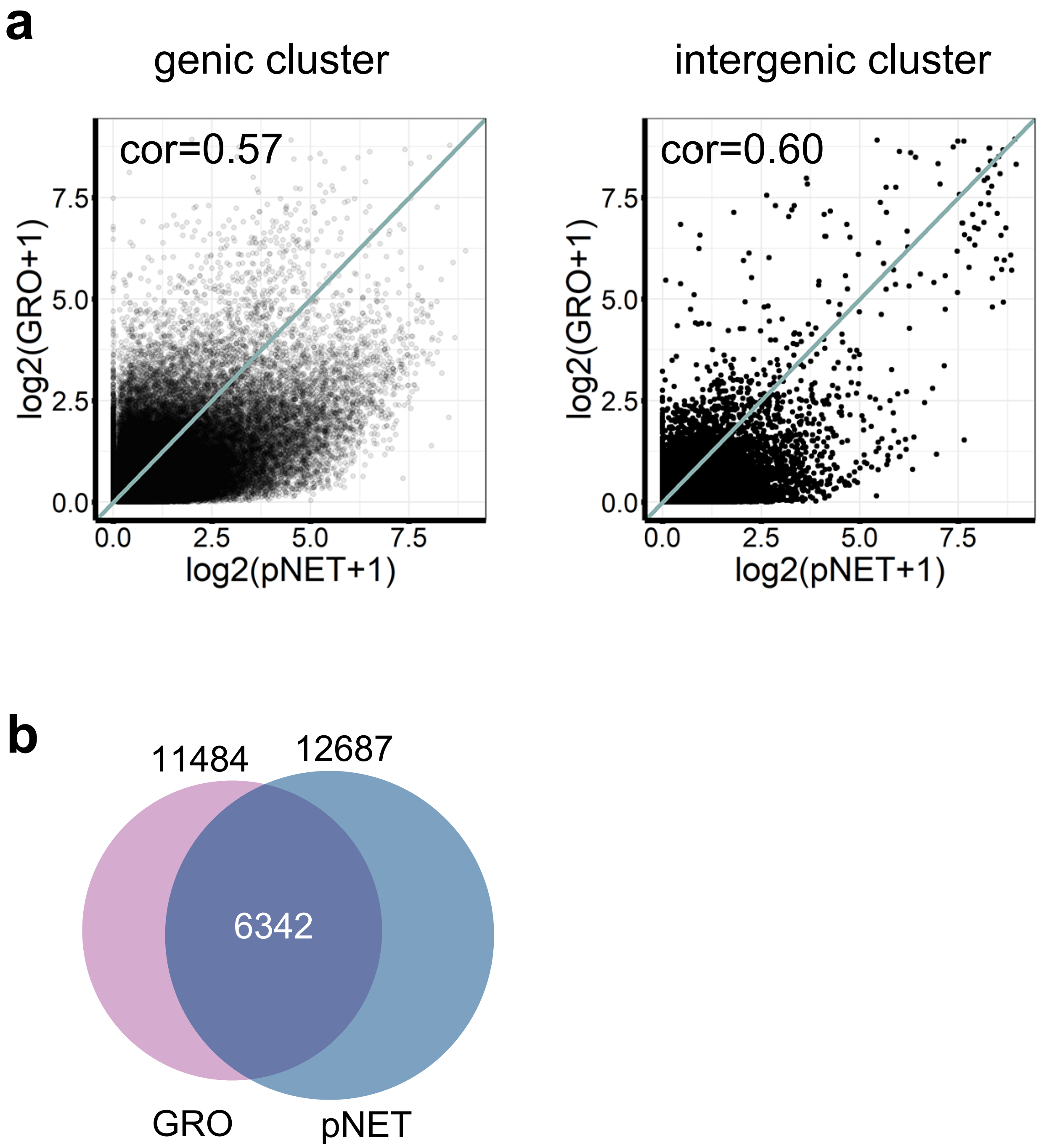

Figure S4. Comparison of GRO-seq and pNET-seq in nascent RNA detection.
a, Correlation of read density of transcription clusters from genic and intergenic regions detected by GRO-seq and pNET-seq, respectively.
b, Venn of intergenic transcription clusters detected by GRO-seq and pNET-seq.

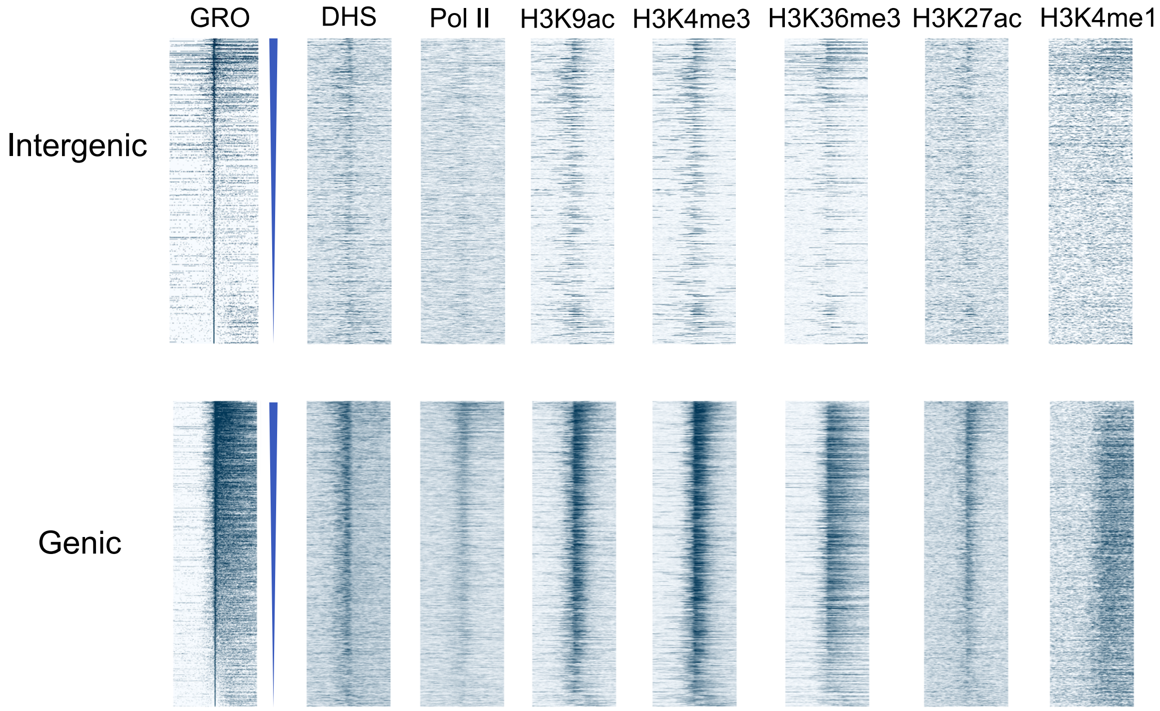

Figure S5. Read densities of GRO-seq, DNase-seq and ChIP-seq of Pol II, H3K9ac, H3K4me3, H3K36me3, H3K27ac, and H3K4me1 around the intergenic and genic transcription clusters (TCs, ± 3kb). All intergenic and genic TCs were ranked in a descending order of GRO-seq signals (±250 bp around the 5' end), and chromatin features were plotted around the 5' end of each intergenic and genic TCs.

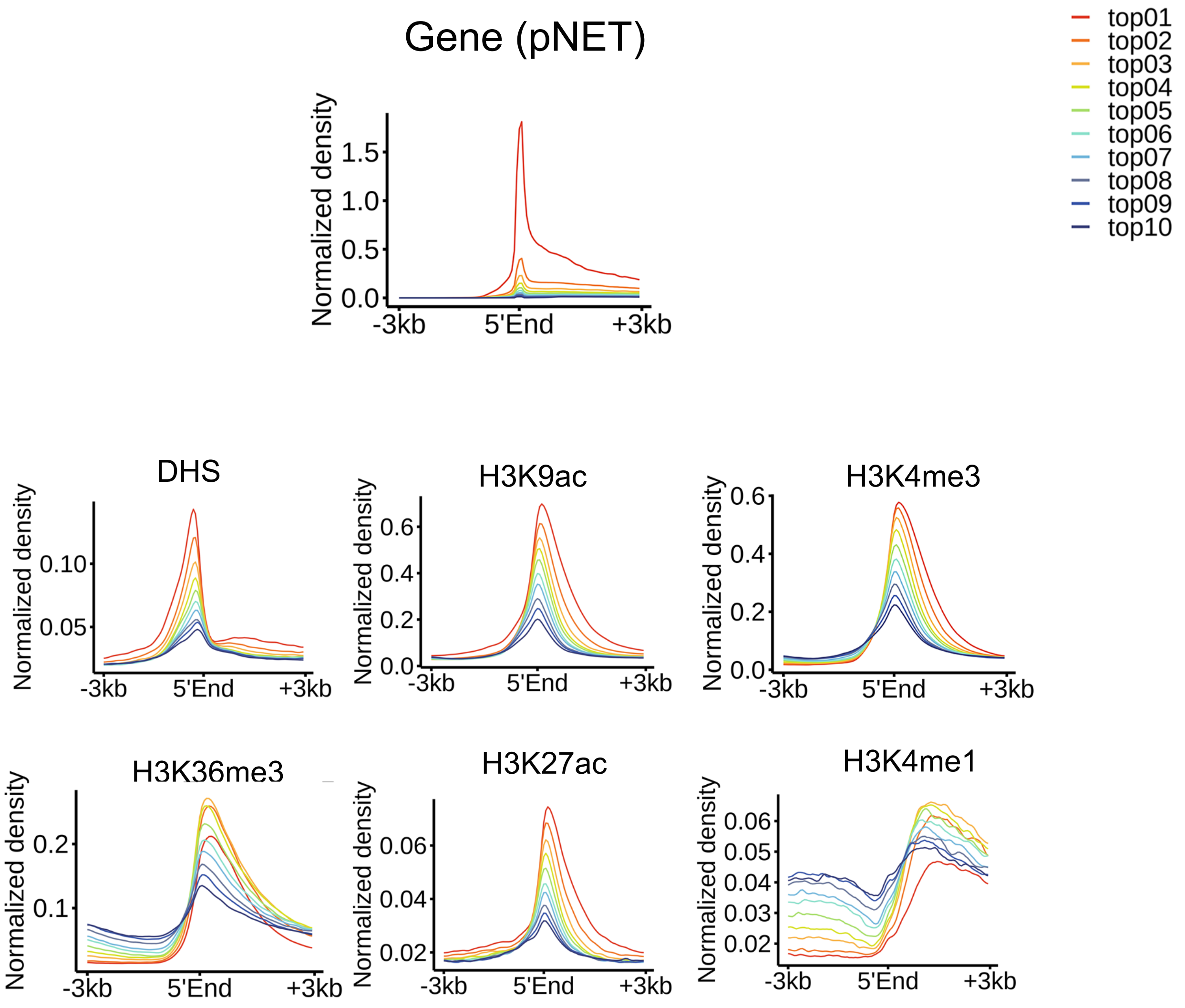

Figure S6. Chromatin states around the genic transcription clusters (TCs) by pNET-seq. Genic TCs were divided into ten equal parts based on the decreasing level of pNET-seq signals (± 250 bp). Read densities of DNase-seq and ChIP-seq of H3K9ac, H3K4me3, H3K36me3, H3K27ac, and H3K4me1 around each of the ten parts of genic TCs.

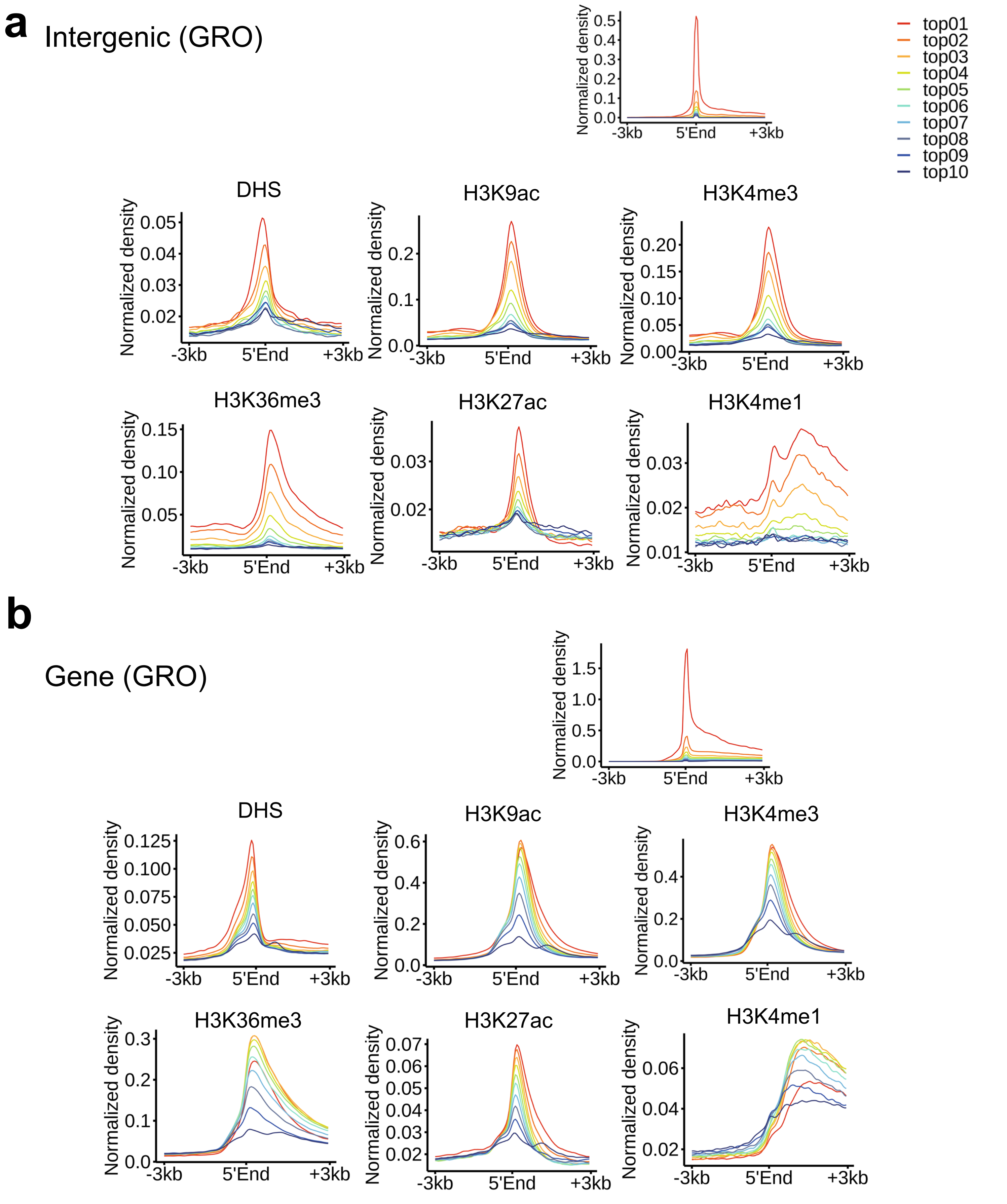

Figure S7. Chromatin states around the intergenic (a) and genic (b) transcription clusters (TCs) by GRO-seq. Intergenic and genic TCs were divided into ten equal parts based on the decreasing level of GRO-seq signals (± 250 bp). Read densities of DNase-seq and ChIP-seq of H3K9ac, H3K4me3, H3K36me3, H3K27ac, and H3K4me1 around each of the ten parts of intergenic and genic TCs.

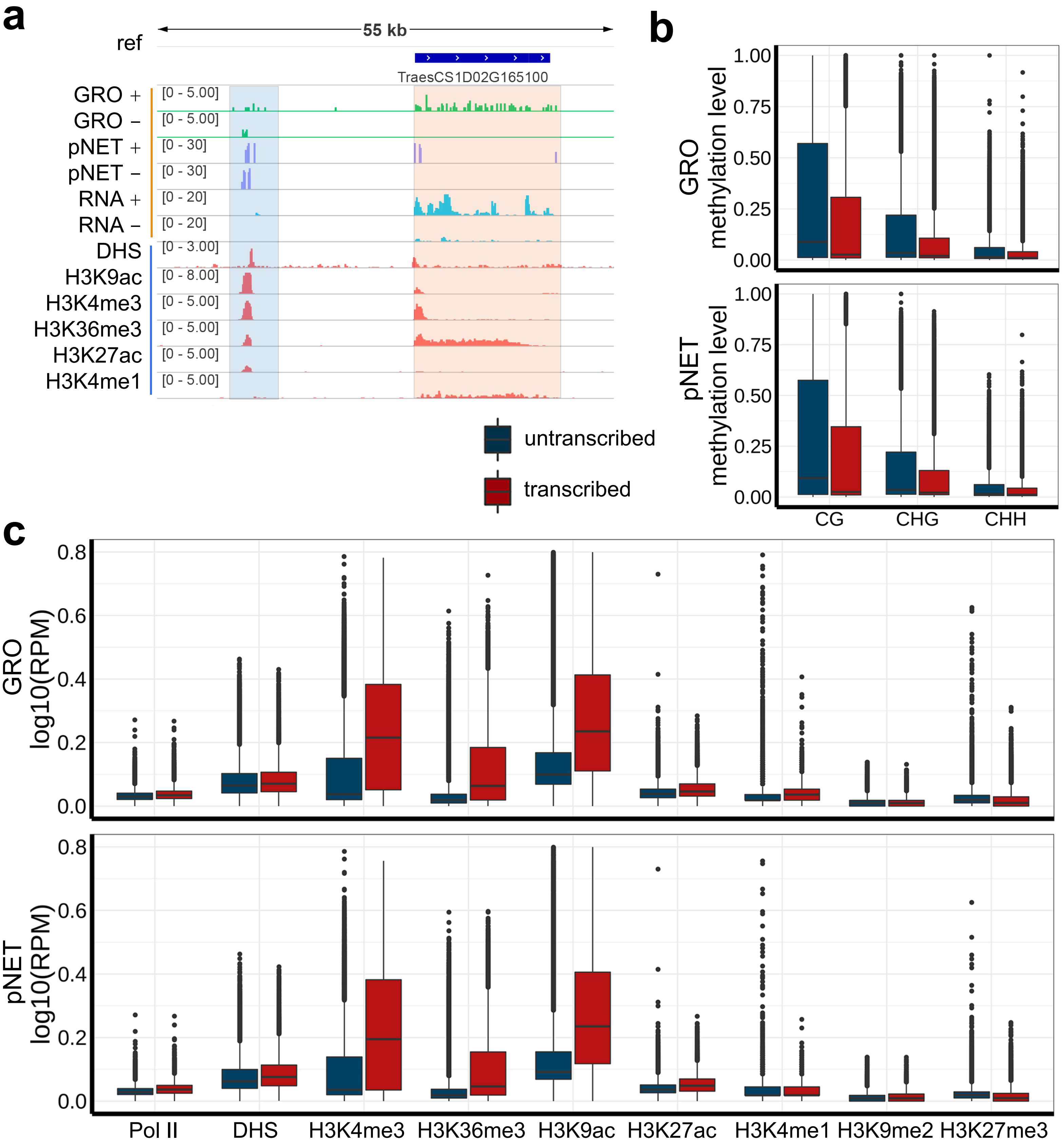

Figure S8. Global identification of intergenic transcribed enhancers in bread wheat.
a, Browser shot of an intergenic transcribed region with enhancer chromatin features (shaded in blue).
b, DNA methylation levels of transcribed and untranscribed enhancers.
c, Chromatin features of transcribed and untranscribed enhancers.
Enhancer transcription detected by GRO-seq and pNET-seq are indicated respectively in b and c.

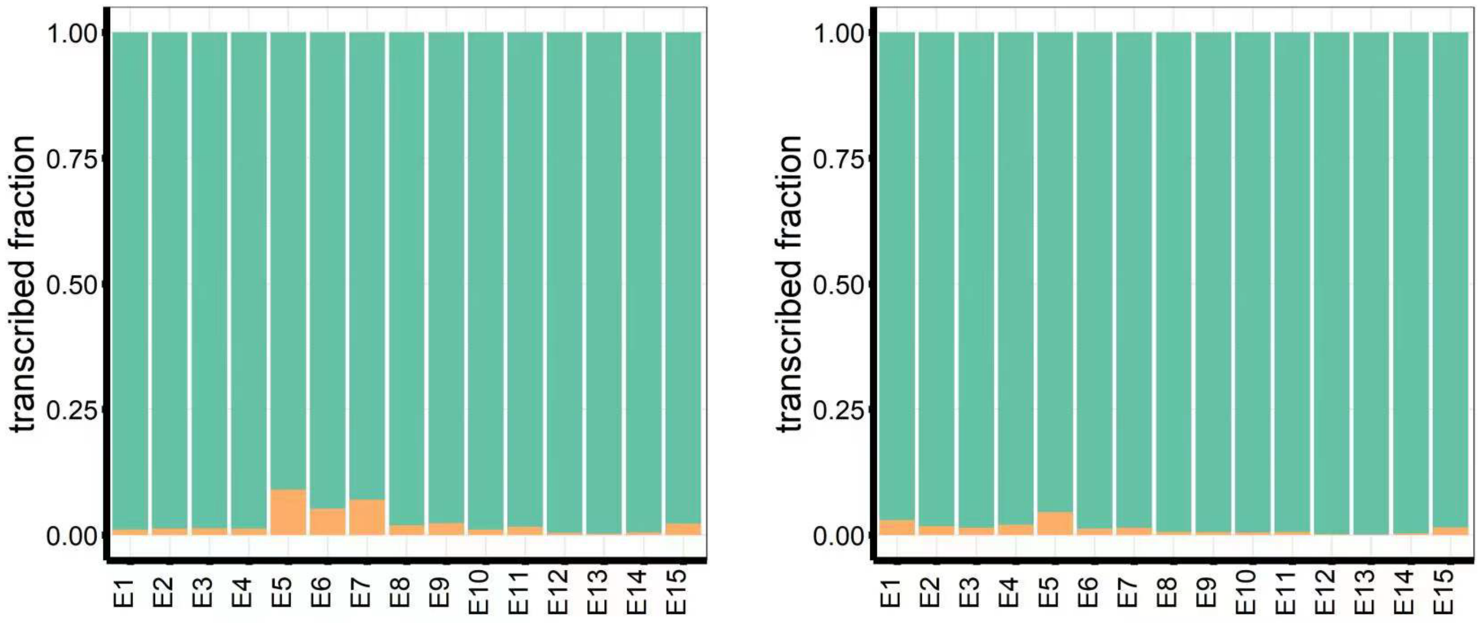

Figure S9. The active transcribed (orange) and non-transcribed (green) fractions in states 1-15 detected by pNET-seq (left panel) and GRO-seq (right panel).

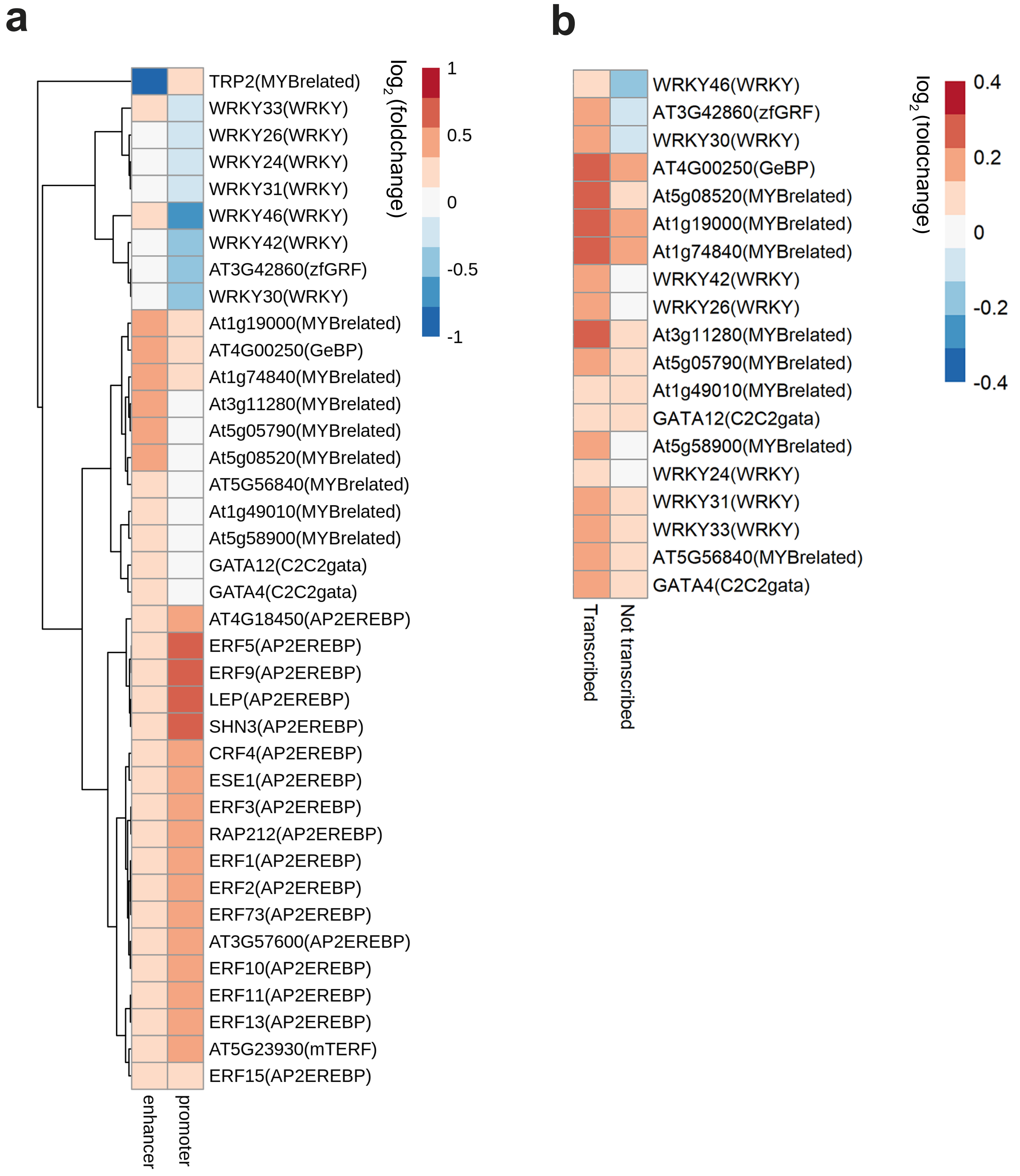

Figure S10. Motif enrichment of promoter and enhancer.

a, Log2(fold change) of the motif occurrence in promoter and enhancer regions relative to the genomic background.

b, Log2(fold change) of the enhancer-enriched-motif occurrence in the transcribed and untranscribed enhancer regions relative to the genomic background.

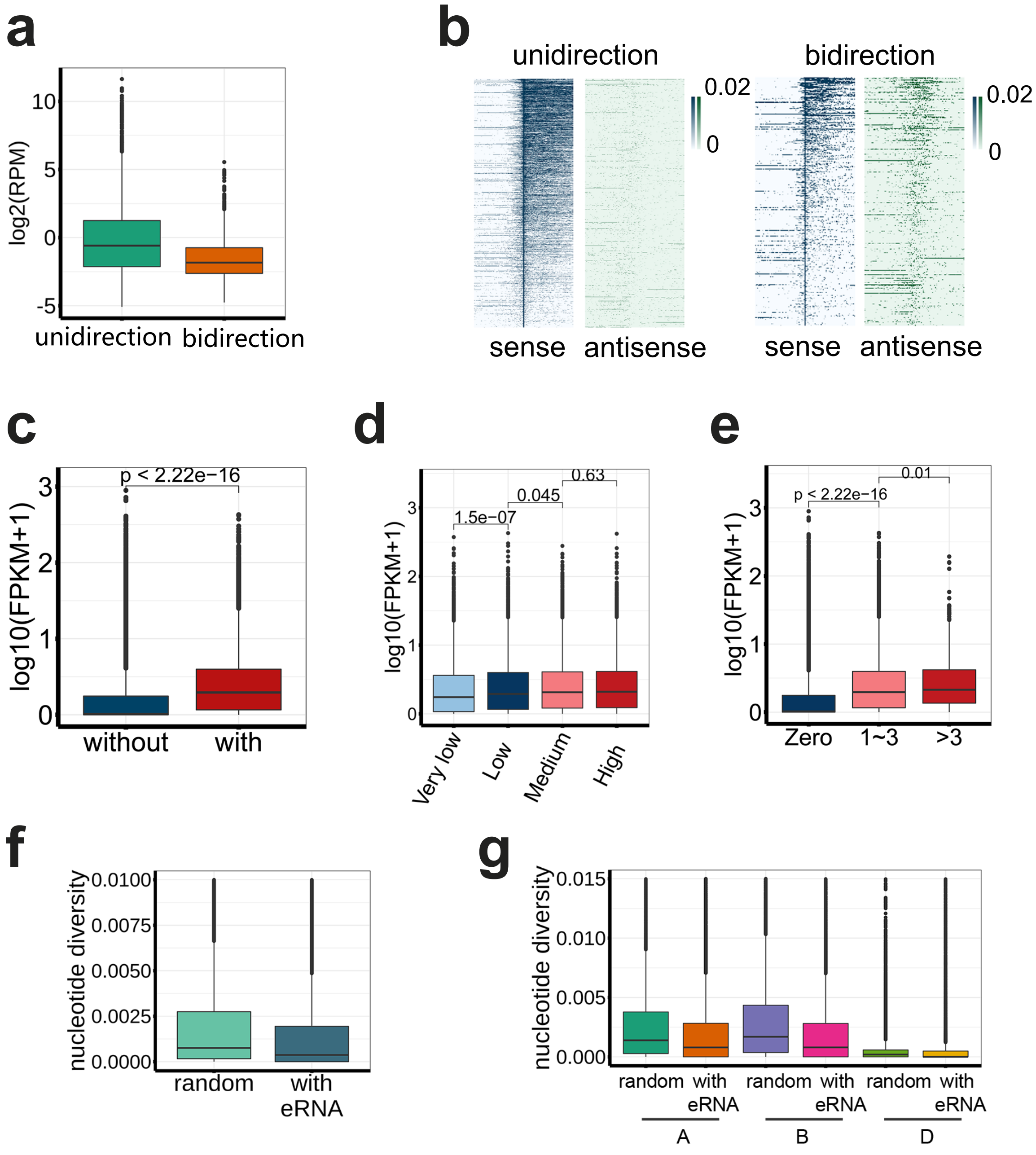

Figure S11. Charaterization of eRNA identified by GRO-seq.

a, Expression levels of unidirectional and bidirectional enhancers.

b, Heatmap display the expression levels of unidirectional and bidirectional enhancers in primary (sense) and/or secondary (antisense) orientations (±3kb).

c, Genes associated with transcribed enhancer display higher expression levels.

d, The enhancer transcription level positively correlates with its target gene expression.

e, The number of associated transcribed enhancers positively correlates with its target gene expression.

f, g, Nucleotide diversity distribution of enhancers with eRNA and random intergenic regions across the genome (f) and within A, B, and D subgenomes (g).

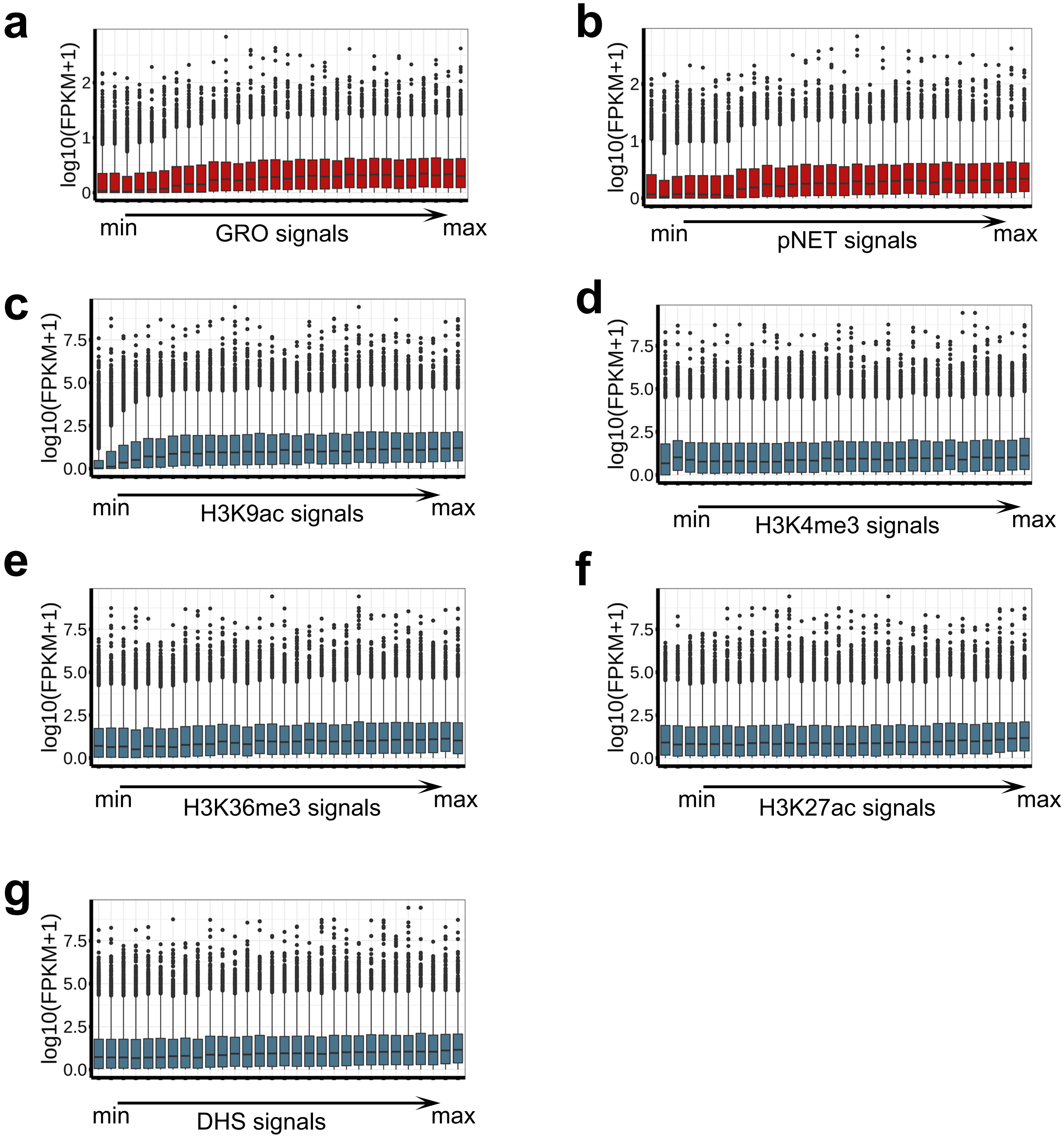

Figure S12. The correlation between enhancer transcription or chromatin states and target gene expression.

a-g, Boxplots showing transcript levels of target genes (by RNA-seq) associated with enhancers with different eRNA levels (a-b), histone modifications (c-f), and chromatin accessibility (g) levels. The target gene expression positively correlates with the associated enhancer transcription level by GRO-seq (a) and pNET-seq (b), and the H3K9ac signals (c), but not with other histone modification (d-f) or chromatin accessibility (g).

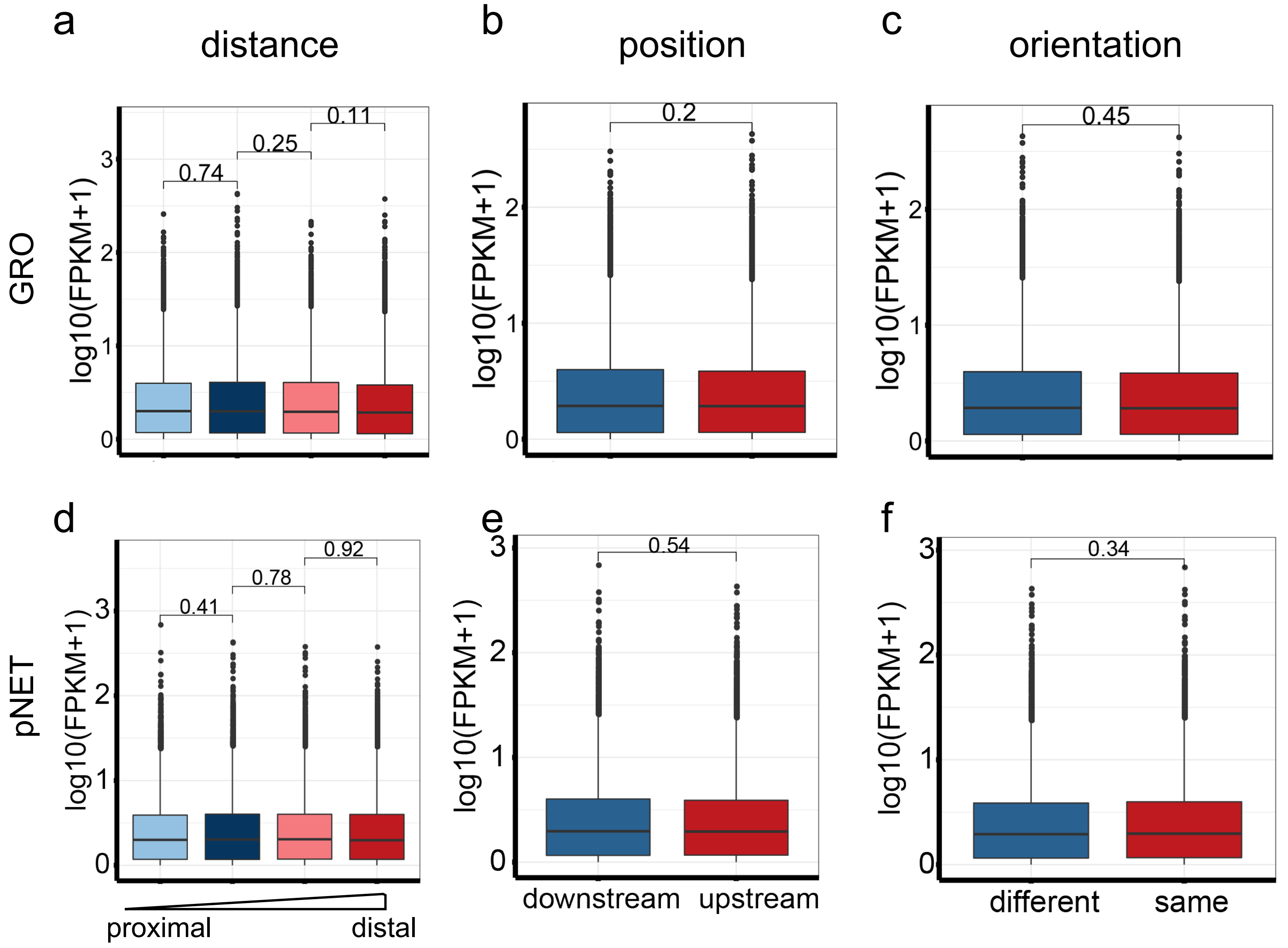

Figure S13. The expression levels of target genes are independent of the distance, position, and orientation of associated transcribed enhances. a-f, Boxplots showing transcript levels of target genes associated with transcribed enhancers with different distances (a, d), positions (b, e), and orientations (c, f). The target gene expression does not correlate with the distance, position, and orientation of associated transcribed enhancers identified by GRO-seq (a-c) or pNET-seq (d-f).

a

b

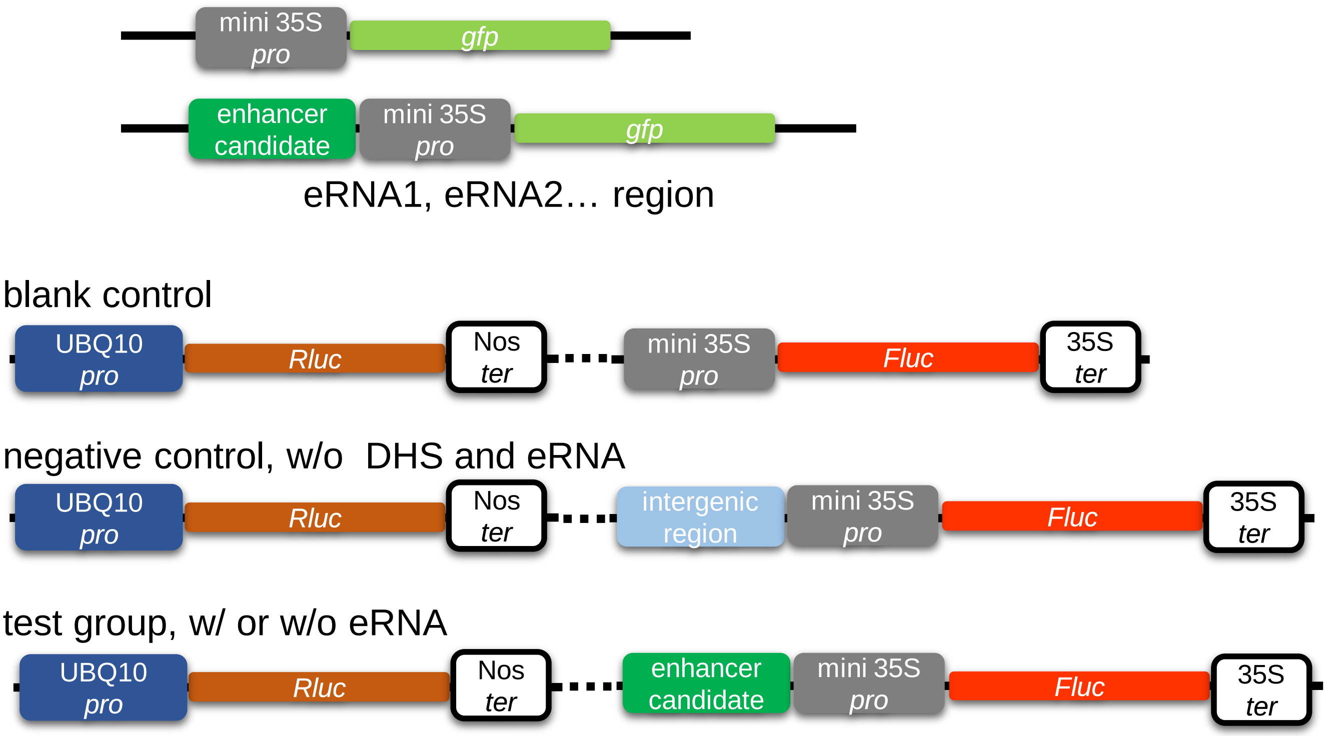

c

	relative expression	enhancer activity (relative intensity)
blank control (B)	$Fluc^B/Rluc^B$	$\frac{Fluc^B/Rluc^B}{Fluc^B/Rluc^B} = 1$
negative control (N)	$Fluc^N/Rluc^N$	$\frac{Fluc^N/Rluc^N}{Fluc^B/Rluc^B} = 1.6$
test group (T)	$Fluc^T/Rluc^T$	$\frac{Fluc^T/Rluc^T}{Fluc^B/Rluc^B} \geq 2.0$ (positive cutoff)

Figure S14. Reporter constructs for enhancer activity measurement. a, Expression cassettes using *gfp* (*green fluorescent protein*) as reporter. b, Expression cassettes using *Rluc* (*Renilla luciferase*) and *Fluc* (*firefly luciferase*) as dual reporters. c, A formula for calculating enhancer activity based on luciferase activity. Blank control, mini 35S pro, a minimal cauliflower mosaic promoter. Negative control, an intergenic region, where there were no DHS, pNET-seq, and GRO-seq signals, fused with the mini 35S pro. Text group, enhancer candidate region fused with the mini 35S pro. For each experiment, *Rluc* driven by a *UBQ10* promoter was used to monitor transfection efficiency in the same vector via the dual-luciferase assays, and the relative expression level was defined as the ratio of Fluc to Rluc. The relative expression level of the test group was normalized to that of the blank control, yielding enhancer activity (relative intensity). Relative intensity ≥ 2.0 was set as the cutoff for the positive enhancer that activated reporter expression.

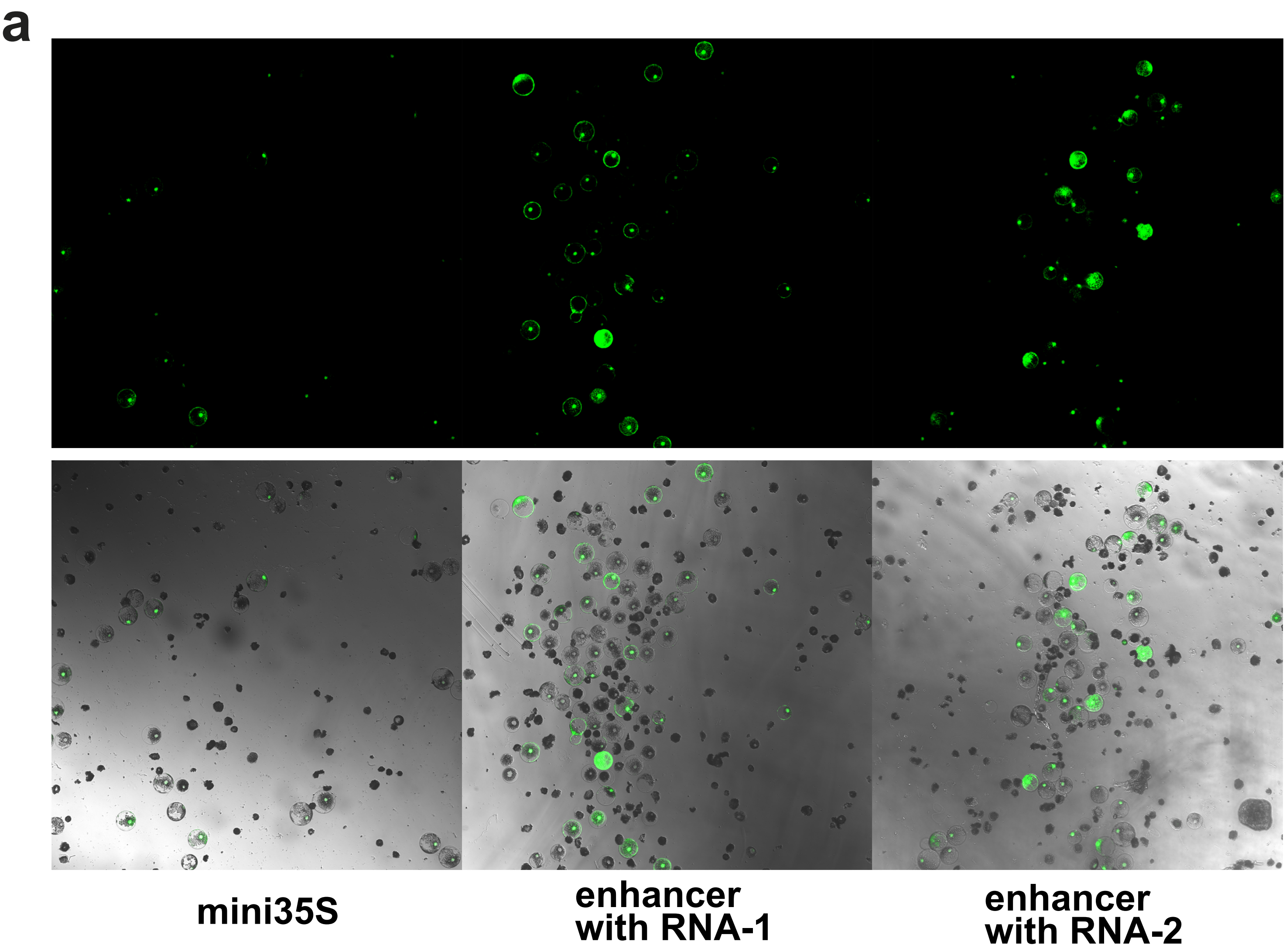

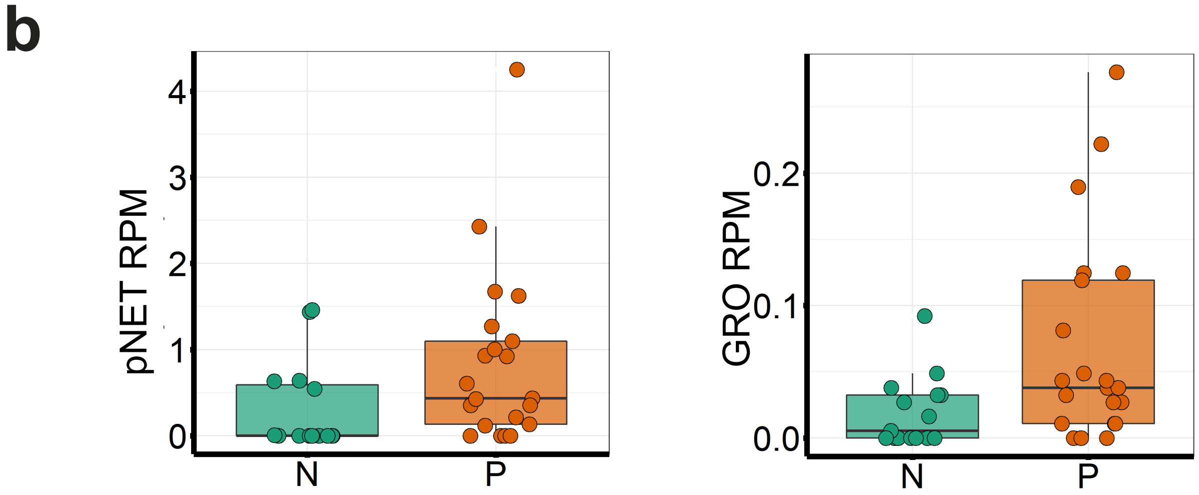

Figure S15. Test the activity of enhancer candidates in wheat protoplasts.
a, Wheat protoplasts transfected with the blank control vector (*mini35Spro:gfp*, left) or vectors with enhancer candidates (*enhancer-mini35Spro:gfp*, middle and right). The GFP signal in the control experiment was weak and confined to the nucleus, whereas those with *enhancer-mini35Spro:gfp* had a bright GFP signal distributed throughout the cell.
b, pNET-seq (left) and GRO-seq (right) signal densities are significant higher in positive (P) than negative (N) candidate enhancers determined in wheat protoplasts (t.test, p=0.049 for pNET-seq and p=0.011 for GRO-seq).

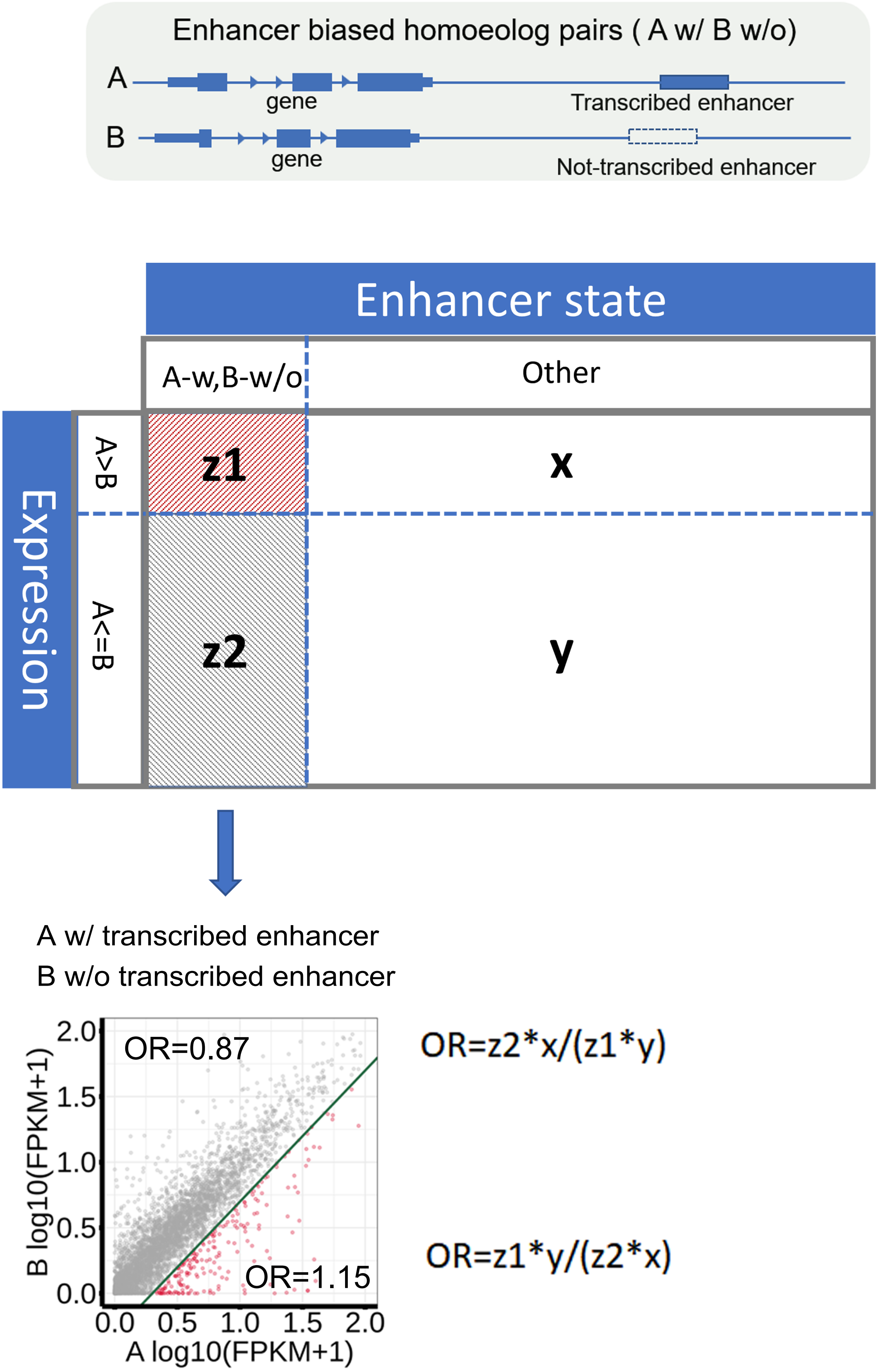
Enhancer biased homoeolog pairs (A w/ B w/o)
A
gene
Transcribed enhancer
B
gene
Not-transcribed enhancer
Enhancer state
A-w,B-w/o
Other
Expression
A>B
A<=B
z1
x
z2
y
A w/ transcribed enhancer
B w/o transcribed enhancer
OR=0.87
OR=1.15
B log10(FPKM+1)
A log10(FPKM+1)
0.0
0.5
1.0
1.5
2.0
OR=z2*x/(z1*y)
OR=z1*y/(z2*x)

Figure S16. Analysis scheme of correlation between subgenome-divergent gene expression and subgenome-biased enhancer transcription.

In most cases, there is a copy of a homeolog gene for each of the three subgenome homeologous sites. These three genes are referred to as a triad. For the sake of correlation analysis, we focused on 67,108 pairs of homoeolog genes that have 1:1 correspondence between any two of the three subgenomes.

Taking the homeolog genes from A and B subgenomes as example, gene pairs can be divided into four scenario: I, homeolog A is associated with transcribed enhancer(s), homeolog B is not; II, homeolog B is associated with transcribed enhancer(s), homeolog A is not; III, both of homeolog A and B are associated with transcribed enhancer(s); IV, neither of homeolog A and B is associated with a transcribed enhancer. We named scenario I as “A W, B W/O” and scenario II-IV as “other”. (In the case that homeolog B is associated with at least one transcribed enhancer, while homeolog A is not, the same statistics were performed. Since A and B are symmetric, only the former case is discussed below.) Based on the expression level, all the gene pairs can be divided into: “A > B” and “A <= B”. Thus, all the gene pairs were divided into four quadrants, z1 (red dots, “A W, B W/O” ∩ “A > B”), z2 (grey dots, “A W, B W/O” ∩ “A <= B”), x, and y.

We used the odds ratio (OR) to measure how strongly “A being expressed higher than B” is associated with “A is associated with transcribed enhancer(s) but B is not”. The odds ratio is a ratio of two sets of odds:

$$\text{odds1}=\frac{\text{A being expressed higher than B when A is associated with transcribed enhancer(s) but B is not (z1)}}{\text{A being expressed no higher than B when A is associated with transcribed enhancer(s) but B is not (z2)}}$$

$$\text{odds2}=\frac{\text{A being expressed higher than B when A is not associated with transcribed enhancer(s) (x)}}{\text{A being expressed no higher than B when A is not associated with transcribed enhancer(s) (y)}}$$

The formula for calculating the odds ratio (OR) are listed aside.

OR > 1 (red dots, odds1/odds2) means that “A is associated with transcribed enhancer(s) but B is not” increase the likelihood of “homeolog A being expressed higher than homeolog B”; while OR < 1 (grey dots, odds2/odds1) means that “A is associated with transcribed enhancer(s) but B is not” decrease the likelihood of “homeolog A being expressed no higher than homeolog B”.

Taken together, it is suggested that biased expression of enhancers associated with homeolog A and B could be a reason for asymmetric expression of homeolog A and B themselves.

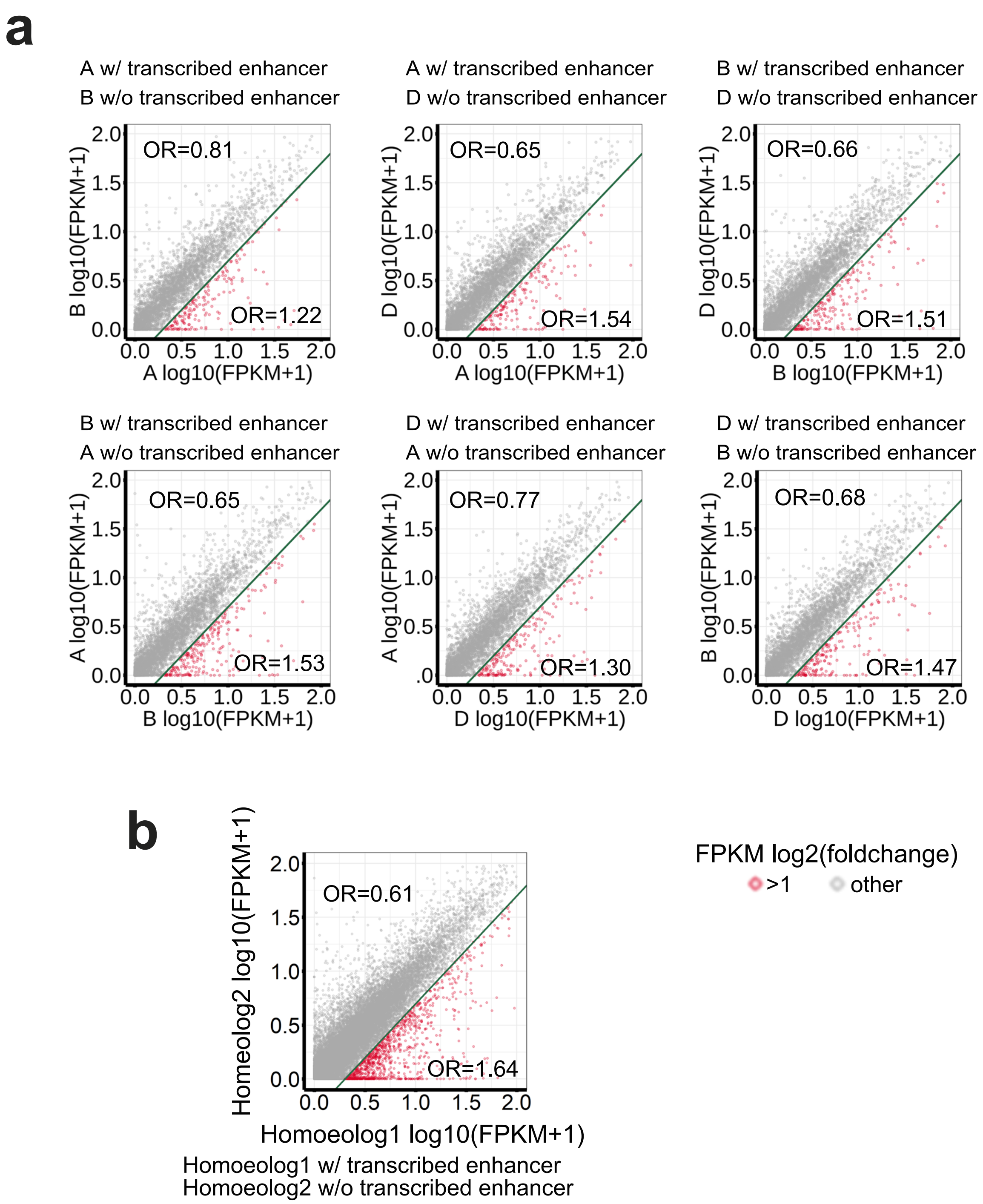

Figure S17. Effects of biased enhancer expression on subgenome gene expression.
a, Subgenome biased transcribed enhancers are enriched with unbalanced expressed homoeologs between each two subgenome pairs (from GRO-seq data). b, Merge all homoeolog pairs in a showed that the dominantly expressed homoeologs essentially associated with transcribed enhancers.

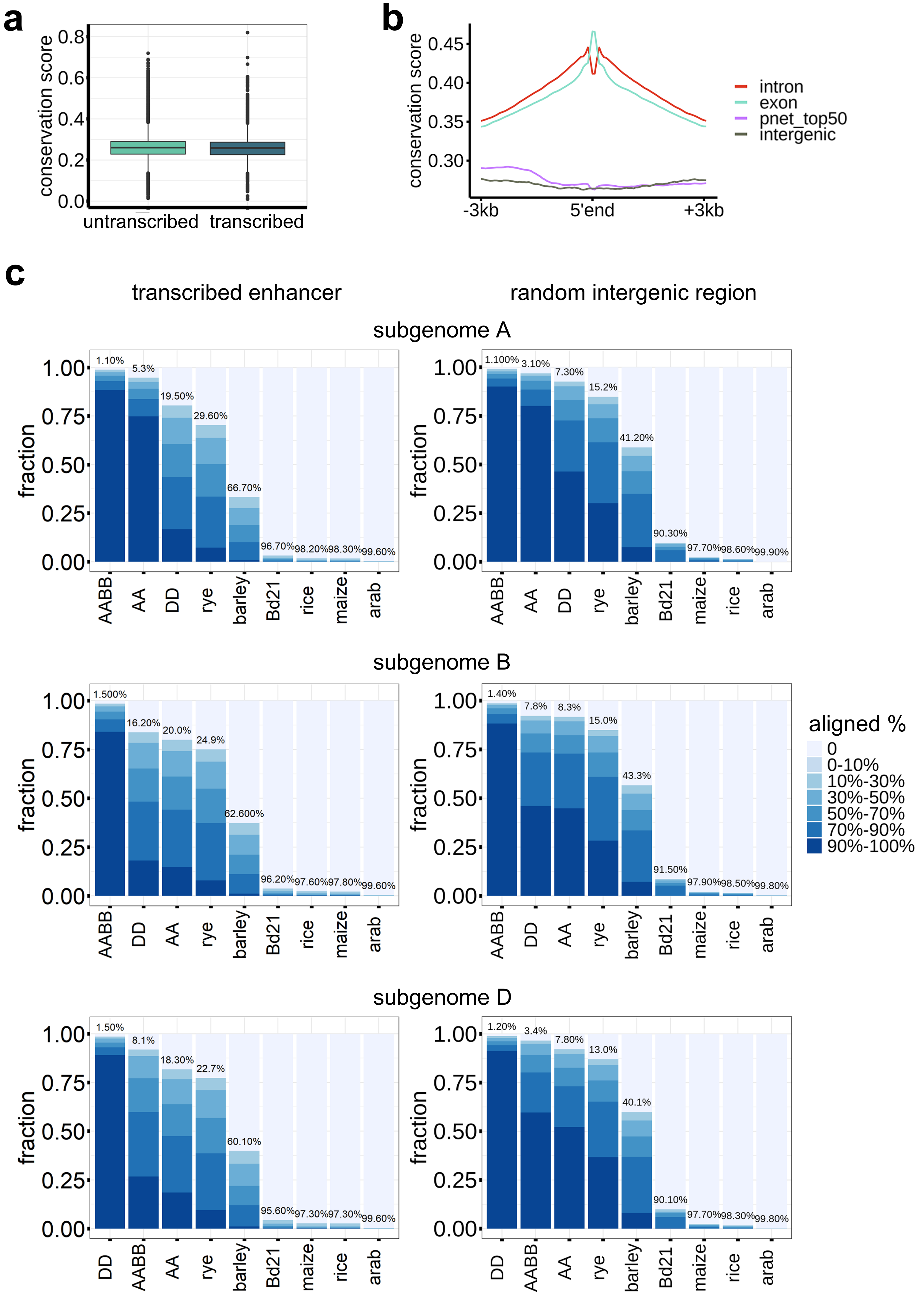
a
conservation score
untranscribed
transcribed
b
conservation score
intron
exon
pnet_top50
intergenic
-3kb
5'end
+3kb
c
transcribed enhancer
random intergenic region
subgenome A
subgenome B
subgenome D
fraction
aligned %
0
0-10%
10%-30%
30%-50%
50%-70%
70%-90%
90%-100%
AABB
AA
DD
rye
barley
Bd21
rice
maize
arab

FigureS18. Homology analysis of eRNA region (by pNET-seq) in wheat and other plants

a, Boxplot show the conservation score distribution in the transcribed and untranscribed enhancer regions. The calculation of the sequence conservation score was described as [1].

b, Conservation score around different type of genomic regions.

c, Distributions of sequence homology between transcribed enhancer region and other species. Minimap2 [67] were used to align the sequence to the genome of each corresponding species. AABB, *Triticum turgidum*; AA, *Triticum Urartu*; DD, *Aegilops tauschii*; rye, *Secale cereale*; barley, *Hordeum vulgare L.*; Bd21, *Brachypodium distachyon*; rice, *Oryza sativa*; maize, *Zea mays*; arab, *Arabidopsis thaliana*. The proportions of aligned regions are presented.

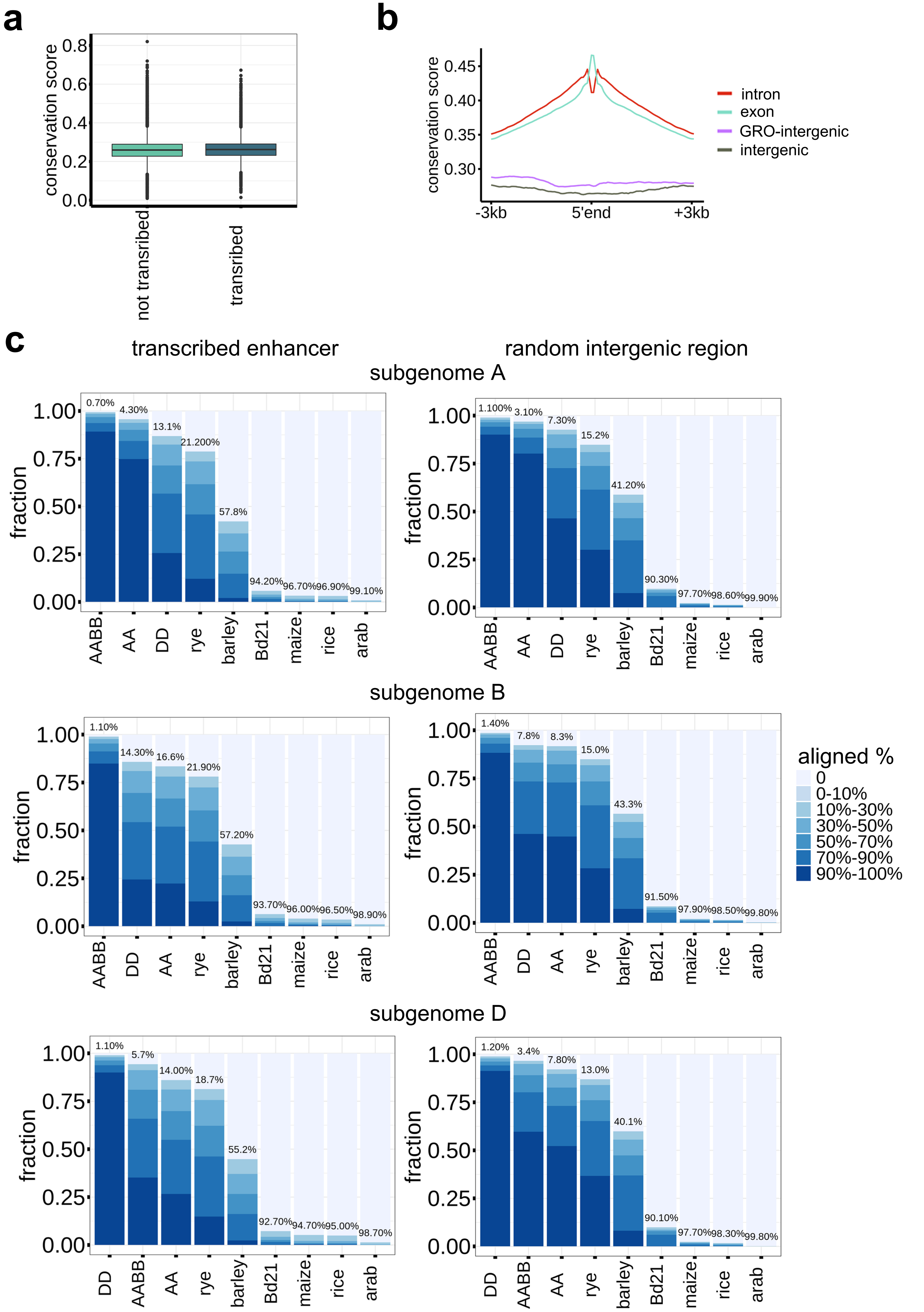
a
conservation score
0.0
0.2
0.4
0.6
0.8
not transribed
transribed
b
conservation score
0.30
0.35
0.40
0.45
-3kb
5'end
+3kb
intron
exon
GRO-intergenic
intergenic
c
transcribed enhancer
random intergenic region
subgenome A
fraction
0.00
0.25
0.50
0.75
1.00
0.70%
4.30%
13.1%
21.200%
57.8%
94.20%
96.70%
96.90%
99.10%
AABB
AA
DD
rye
barley
Bd21
maize
rice
arab
1.100%
3.10%
7.30%
15.2%
41.20%
90.30%
97.70%
98.60%
99.90%
subgenome B
1.10%
14.30%
16.6%
21.90%
57.20%
93.70%
96.00%
96.50%
98.90%
AABB
DD
AA
rye
barley
Bd21
maize
rice
arab
1.40%
7.8%
8.3%
15.0%
43.3%
91.50%
97.90%
98.50%
99.80%
aligned %
0
0-10%
10%-30%
30%-50%
50%-70%
70%-90%
90%-100%
subgenome D
1.10%
5.7%
14.00%
18.7%
55.2%
92.70%
94.70%
95.00%
98.70%
DD
AABB
AA
rye
barley
Bd21
maize
rice
arab
1.20%
3.4%
7.80%
13.0%
40.1%
90.10%
97.70%
98.30%
99.80%

FigureS19. Homology analysis of eRNA region (by GRO-seq) in wheat and other plants

a, Boxplot show the conservation score distribution in the transcribed and untranscribed enhancer regions. The calculation of the sequence conservation score was described as [1].

b, Conservation score around different type of genomic regions.

c, Distributions of sequence homology between transcribed enhancer region and other species. Minimap2 [67] were used to align the sequence to the genome of each corresponding species. AABB, *Triticum turgidum*; AA, *Triticum Urartu*; DD, *Aegilops tauschii*; rye, *Secale cereale*; barley, *Hordeum vulgare L.*; Bd21, *Brachypodium distachyon*; rice, *Oryza sativa*; maize, *Zea mays*; arab, *Arabidopsis thaliana*. The proportions of aligned regions are presented.

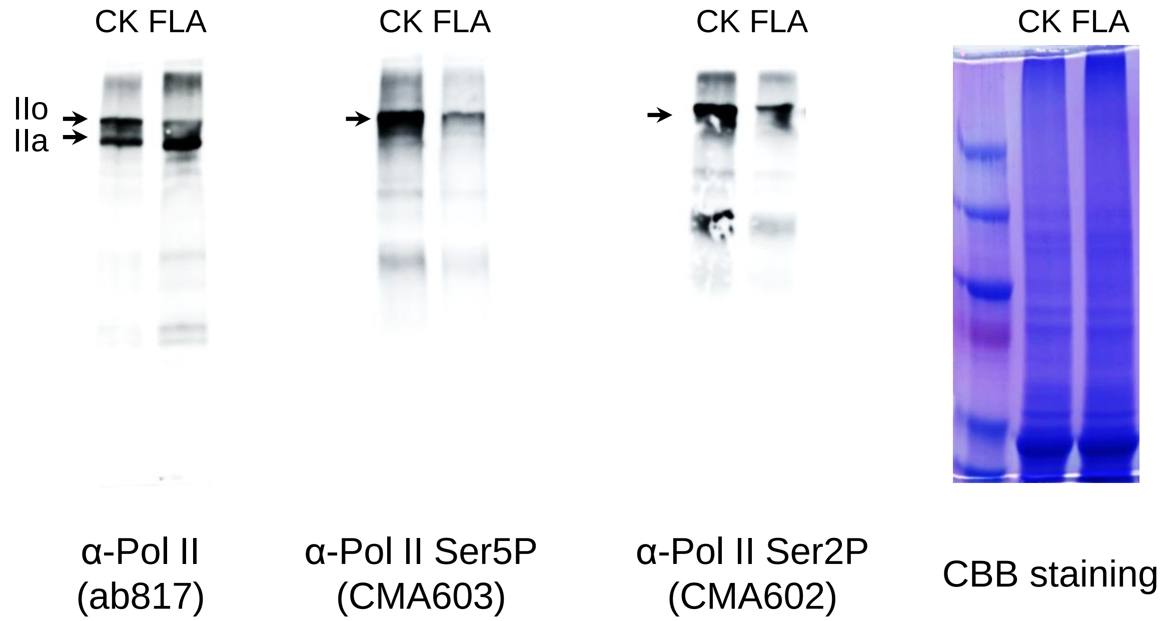

FigureS20. Antibody (ab817) used for pNET-seq can recognize the both unphosphorylated and phosphorylated CTD isoforms of wheat RNA polymerase II. Immunoblots of Pol II CTD phosphorylation in control and seedlings after a 2-hour treatment with flavopiridol (FLA). FLA has been shown to inhibit phosphorylation of Pol II CTD heptamer repeats at Ser2 and Ser5 in Arabidopsis. After FLP treatment, the signal intensity by CMA602 (Ser2P specific) and CMA603 (Ser5P specific) decreased, suggesting that FLP block Pol II phosphorylation in wheat seedling as well. Two bands were labelled by ab817, while the upper band was decreased by FLA treatment. Taken together, ab817 can detect unphosphorylated and phosphorylated CTD isoforms of wheat Pol II. Coomassie Brilliant Blue staining (CBB staining) was used as a loading control. IIo and IIa indicate phosphorylated and unphosphorylated Pol II.

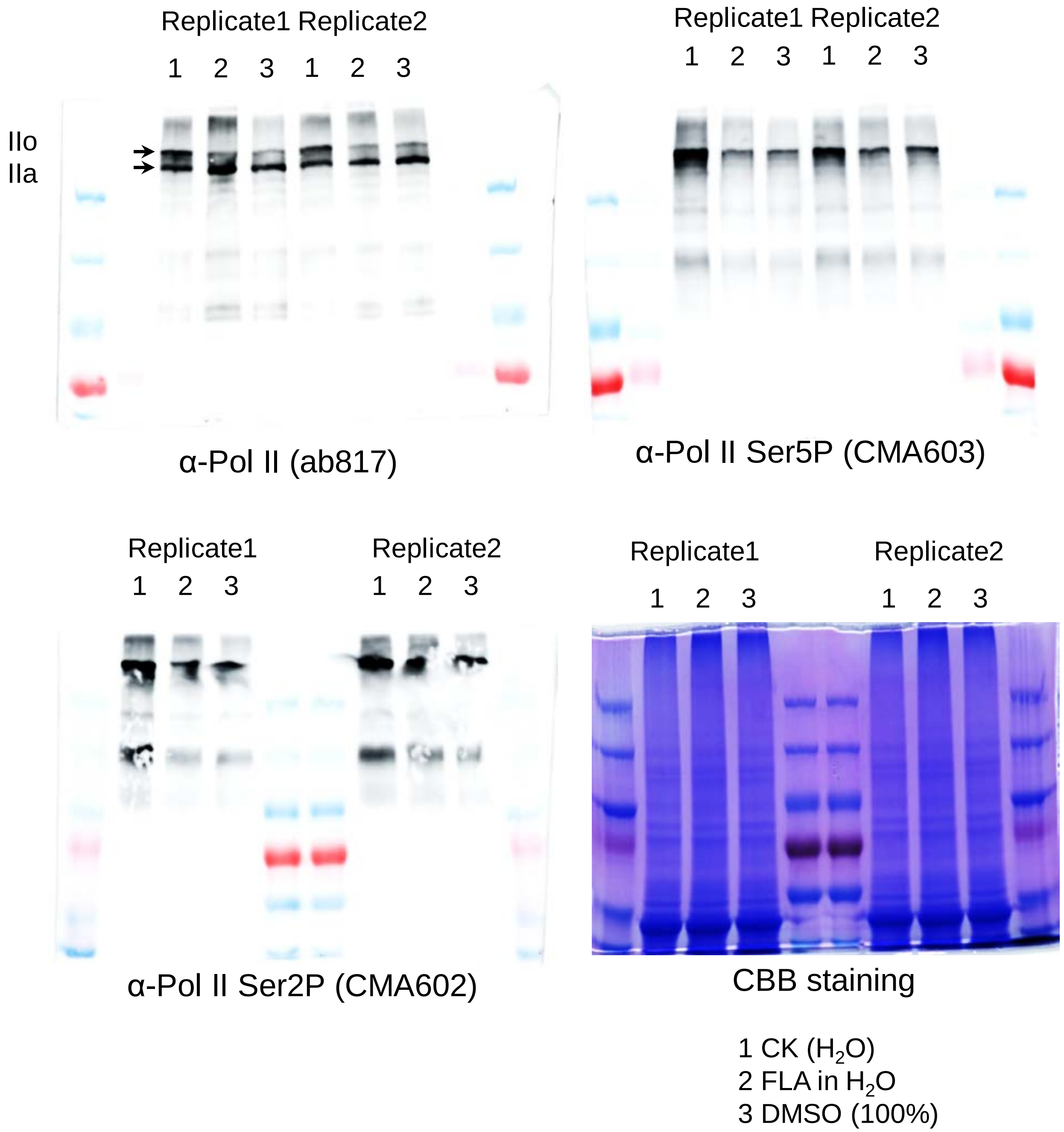

FigureS21. Uncropped images for FigureS20.

Immunoblots of Pol II CTD phosphorylation in control and seedlings after a 2-hour treatment with flavopiridol (FLA). FLA has been shown to inhibit phosphorylation of Pol II CTD heptamer repeats at Ser2 and Ser5 in Arabidopsis. After FLP treatment, the signal intensity by CMA602 (Ser2P specific) and CMA603 (Ser5P specific) decreased, suggesting that FLP block Pol II phosphorylation in wheat seedling as well. Two bands were labelled by ab817, while the upper band was decreased by FLA treatment. Taken together, ab817 can detect unphosphorylated and phosphorylated CTD isoforms of wheat Pol II. Notably, 100% DMSO also inhibits phosphorylation of Pol II CTD. Coomassie Brilliant Blue staining (CBB staining) was used as a loading control. IIo and IIa indicate phosphorylated and unphosphorylated Pol II.
