# Supplementary material for: Enhancer transcription detected in the nascent transcriptomic landscape of bread wheat
Source: Genome Biol. 2022 Apr 30;23:109. doi: 10.1186/s13059-022-02675-1 (PMC9063354; doi:10.1186/s13059-022-02675-1)
Supplement: Supplementary file 8 — Additional file 8. Review history. [file 13059_2022_2675_MOESM8_ESM.docx]

Review History

**First round of review**

**Reviewer 1**

**Were you able to assess all statistics in the manuscript, including the appropriateness of statistical tests used?**

No, I do not feel adequately qualified to assess the statistics.

**Comments to author:**

In this manuscript, Xie and co-authors reported the transcriptional and epigenomic features of putative enhancers in bread wheat. By using pNET-seq and GRO-seq, the author provided an overview of transcriptional activities in wheat genome. The author found intergenic regions are frequently transcribed and a significant proportion of these are associated with putative enhancers that were annotated previously. They further established a link between the enhancer transcription and the expression of associated genes. Finally, they discussed the relationship between enhancer transcription and sub-genome-biased gene expression. Overall, the work represent important addition to the understanding of wheat genome, as well as for enhancers and transcription field in plant. In the meantime, there are a few outstanding questions needs to be addressed as details below.

1. Throughout the manuscript, the definition of enhancers seems not so clear. I understand the authors defined putative enhancers in a previous work, but the standard they use are equally important for this work, the author should explain it clearly in this work.

2. Line90 to 97. The author described epigenomic features associated with intergenic transcribed region. It is unclear to what extent these features are specific to intergenic region. A side-by-side comparison between gene and intergenic region should be shown.

3. Related to the point above, Figure 1e,f plots the histone modification levels and found they are generally all related to pNET-seq signals. The way of plotting is problematic. I assume in each group that defined by pNET-seq signals, there is a proportion of peaks that do not have histone modification. Inclusion of these peaks when plotting histone modifications could generate artifacts. I suggest the author to re-plot histone modification in each group by removing peaks that do not harbor such modifications. The important thing is whether the pattern still holds after this filtering.

4. The author seems only used pNET-seq data for the majority of the analysis (start from figure 1e). The underlying assumption is pNET-seq data represent active transcription. There are two issues regarding to this.

a. pNET-seq signal could represents paused Pol II, how does the author distinguish paused Pol II and elongating pol II from the data? In this sense, using GRO-seq data is more reasonable. Would GRO-seq data generate same or similar results as the pNET-seq data for enhancer analysis ? The author should test this and present the result to further solid their conclusions.

b. It is unclear to this reviewer about the specificity of Pol II antibody that was used in this study. Does it recognize S2P Pol II in wheat? At the least, a western blot should be provided to show the antibody specificity. In theory, S2P Pol II pNET-seq data should be used if the author equals pNET-seq signals to active transcription.

5. Line 176 to 182, the author looked the sub-genome divergent gene expression in relation to sub-genome-biased enhancer transcription. The explanation of the data is simply too brief for readers to understand it. I found the data in Figure 2i is not strong enough to draw a big conclusion (for example, in abstract "sub-genome-biased enhancer transcription is a potential driving force for sub-genome-biased gene expression"), as the majority of enhancers in these plot are not transcribed (gray dot), so a predominant contribution of enhancer transcription in sub-genome-divergent gene expression is not well supported by data. More analysis should be done to clarify this issue.

Minor point:

1. The colors in Figure 1a is too similar between each features. More contrasting colors should be used.

**Reviewer 2**

**Were you able to assess all statistics in the manuscript, including the appropriateness of statistical tests used?**

Yes, and I have assessed the statistics in my report.

**Comments to author:**

Combining nascent RNA sequencing and epigenome profiling, the authors identified a quarter of "epigeneticly defined enhancers" showing active transcription in bread wheat genome. They also found that the transcriptional enhancers were associated with active epigenetic makers and higher gene expression levels, indicating that enhancer transcription can potentially be used as a hallmark in regulation of plant gene expression. The prediction of regulatory elements based on enhancer transcription is interesting as it may be complementary to the widely used ATAC-seq, DNase-seq, and MH-seq methods.

Major comments:

This would be the first paper demonstrating transcription of active enhancers in plants. Thus, a conclusive wet lab validation of the predicted enhancers should be absolutely required, especially for a high profile journal like GB. I do not agree that a transient and heterologous transcription system is acceptable for such a validation. Wheat and tobacco belong to monocot and dicot plant species, respectively. It is not acceptable to examine the function of a wheat enhancer in such a distantly related plant species for a concept-establishing manuscript. The authors need to use transgenic assays in wheat or CRISPR/cas based deletion assays to validate a subset of wheat enhancers predicted based on transcription.

Minor comments:

Library constructions for GRO-seq and pNET-seq are tricky especially in plants. The manuscript lacks evidence to prove the data quality of GRO-seq and pNET-seq. (a) Does most of the enhancer transcription overlap between the replicates of GRO-seq or pNET-seq data? (b) Does most of the enhancer transcription overlap between GRO-seq and pNET-seq data? (c) Since the nascent RNA transcription was detected, why the numbers of reads mapped to exon and intron regions were so different in Figure 1a? (d) How the "TSS 1k" was defined in Figure 1a? Why the proportion of the reads mapped to "TSS 1k" was only ~5%, considering "transcriptional pausing was observed in close proximity to active transcription start sites".

Are the proportions of intergenic enhancers producing transcripts in state 5-7 significantly higher than other state groups? (page5: line 108-110)

The authors appear to fully ignore the recent plant enhancer research based on open chromatin-mediated prediction. The authors need to review these recent and relevant research work.

**Authors Response**

**Point-by-point responses to the reviewers’ comments:**

Reviewer #1:

In this manuscript, Xie and co-authors reported the transcriptional and epigenomic features of putative enhancers in bread wheat. By using pNET-seq and GRO-seq, the author provided an overview of transcriptional activities in wheat genome. The author found intergenic regions are frequently transcribed and a significant proportion of these are associated with putative enhancers that were annotated previously. They further established a link between the enhancer transcription and the expression of associated genes. Finally, they discussed the relationship between enhancer transcription and sub-genome-biased gene expression. Overall, the work represent important addition to the understanding of wheat genome, as well as for enhancers and transcription field in plant. In the meantime, there are a few outstanding questions needs to be addressed as details below.

*Reply: We appreciated the critical comments and constructive suggestions from reviewer#1.*

1. Throughout the manuscript, the definition of enhancers seems not so clear. I understand the authors defined putative enhancers in a previous work, but the standard they use are equally important for this work, the author should explain it clearly in this work.

*Reply: Thanks for the suggestion. We have added explanation for enhancers annotation “Previously we categorized wheat genome into 15 chromatin states based on combinatorial histone modification marks, using multivariate hidden Markov model (HMM) which has been applied in detection of regulatory elements related to individual marks. Chromatin state 5-7 was marked by high DNA accessibility, histone acetylation, CG island, and relative conserved sequence. Therefore, we defined the chromatin state 5-7 regions, which are 3 kb distant from the nearest gene, as enhancer-like elements. In a tobacco transient system, around half of the putative enhancer-like elements increase the expression of reporter gene.” Lines 112-119.*

2. Line90 to 97. The author described epigenomic features associated with intergenic transcribed region. It is unclear to what extent these features are specific to intergenic region. A side-by-side comparison between gene and intergenic region should be shown.

*Reply: Thanks for the suggestion. We have plotted the meta-profiles of epigenetic features around the genic transcription as well. The results have been included in the Figure 1d and FigureS5.*

*We also modified the description in the text as “We next characterized the epigenomic features surrounding intergenic and genic transcribed regions. Overall, both intergenic and genic transcriptions are strongly associated with open chromatin regions, RNA Pol II engagement and transcriptionally active histone marks including H3K9ac, H3K27ac, H3K4me3, and H3K36me3. However intergenic transcriptions have little or no association with H3K4me1, H3K9me2 and H3K27me3 in comparison to genic regions”. Lines 91-96.*

*Figure for response. Read densities of GRO-seq, pNET-seq, DNase-seq (DHS) and ChIP-seq of Pol II, H3K9ac, H3K4me3, H3K36me3, H3K27ac, H3K4me1, H3K9me2, and H3K27me3 around intergenic and genic TSS clusters (TCs) defined by pNET-seq/GRO-seq (± 3kb). TCs are sorted based on the decreasing level of pNET-seq/GRO-seq signals (± 250 bp around the 5’ end TSS).*

3. Related to the point above, Figure 1e,f plots the histone modification levels and found they are generally all related to pNET-seq signals. The way of plotting is problematic. I assume in each group that defined by pNET-seq signals, there is a proportion of peaks that do not have histone modification. Inclusion of these peaks when plotting histone modifications could generate artifacts. I suggest the author to re-plot histone modification in each group by removing peaks that do not harbor such modifications. The important thing is whether the pattern still holds after this filtering.

*Reply: Thanks for the critical comments. We re-plotted the histone modification profiles by removing the TSSs that do not harbor such modifications. DHS, H3K9ac and H3K4me3 still hold the patterns as these three modifications are the major markers of enhancer-like elements. To be cautious, we left out the inference on H3K27ac, H3K4me1, H3K9me2, and H3K27me3. Lines 100-106.*

*Figure for response. Read densities of DNase-seq and ChIP-seq of H3K9ac, H3K4me3, H3K36me3, H3K27ac, H3K4me1, H3K9me2, and H3K27me3 around 5’ end in each of the ten parts of intergenic TCs. The TCs which do not have histone modification are removed.*

*However, we respectfully disagree with the reviewer that peaks without histone modifications should be removed. For a chromatin location, even if there is no histone modification, it should be considered as a chromatin state in the analysis. If those peaks without histone modification were excluded from the meta-profiling, the results would certainly be biased toward to high histone modifications and only reflect part of the whole picture. Because effects of histone modifications on gene expression were better understood than intergenic regions, we compared the histone modification meta-profiles using all the genes and genes with certain histone modification. For example, H3K9me2 (as a repressive mark for TE or repetitive sequence) has little correlation with the nascent RNA for all gene set (below, middle panel), while it is somehow enriched in certain expression percentiles of genes when no H3K9me2 genes were removed (below, right panel). Notably, the normalized density is quiet high in the genes with H3K9me2. We think the meta profiles with all genes better reflects the pattern in the heat map (below, left panel). Furthermore, if we remove the non-histone modification sites for each meta-analysis, the selected peaks differ for different modifications and the meta-analyses of different modifications cannot be compared.*

*Figure for response. Comparison of H3K9me2 meta profiles of all genes and genes with H3K9me2.*

4. The author seems only used pNET-seq data for the majority of the analysis (start from figure 1e). The underlying assumption is pNET-seq data represent active transcription. There are two issues regarding to this.

a. pNET-seq signal could represents paused Pol II, how does the author distinguish paused Pol II and elongating pol II from the data? In this sense, using GRO-seq data is more reasonable. Would GRO-seq data generate same or similar results as the pNET-seq data for enhancer analysis ? The author should test this and present the result to further solid their conclusions.

b. It is unclear to this reviewer about the specificity of Pol II antibody that was used in this study. Does it recognize S2P Pol II in wheat? At the least, a western blot should be provided to show the antibody specificity. In theory, S2P Pol II pNET-seq data should be used if the author equals pNET-seq signals to active transcription.

*Reply: Thanks for the critical comment. In the original analyses, we focused on the pNETseq data because: 1) intergenic transcription were detected by both pNET-seq and GRO-seq, and 2) we obtained much more valid data (unique mapped reads) by pNETseq then. To further solidify the conclusions, we re-did the GRO-seq experiment and obtained a total of 8 times more data than the original one. To get more valid genic and intergenic transcription data at a reasonable sequencing cost, we added rRNA removal step during the GRO-seq library preparation. After rRNA removal, the unique mapping rate was increased from around 1% to 16% and therefore reduced the cost of sequencing by more than ten times. The rRNA removal step has little effect on the quantification of transcriptional activity. The correlation coefficients of GRO-seq replicates were increased from 0.57 to 0.99. And we detected 6,671 more intergenic active transcription clusters. We think this rRNA removal step greatly improves the feasibility of GRO-seq for studying complex genome transcription. In this revision, we did analyses for both pNET-seq and GRO-seq data.*

*We added the description in the Methods section as “We performed two rounds of GRO-seq. For the first time (rep1 and rep2), the ratio of unique mapping reads (we used for further transcription analyses) to total raw reads was 1% (Additional file 2: Supplementary Table 1). For the second time (v2_rep1, v2_rep2, and v2_rep3), to obtain a deeper sequencing coverage at a reasonable sequencing cost, we added a rRNA removal step after nucleus RNA isolation and before the affinity purification of nascent RNA using riboPOOL kit (siTOOLs BIOTECH). Then the unique mapping ratio was increased to 16% (Additional file 2: Supplementary Table 1). The cDNA libraries were sequenced on the Illumina NovaSeq platform. The two rounds GRO-seq data were then merged for further analyses”. Lines 243-252.*

*The analyses on GRO-seq data come up with similar findings as pNET-seq. The overall correlation of transcription clusters between GRO-seq and pNET-seq are 0.57 and 0.60 at genic and intergenic regions, respectively. The epigenetic features are similar around the genic and intergenic transcribed regions called by GRO-seq or pNET-seq (see above). However, we cannot rule out that some of the pNET-seq signal are from paused Pol II rather than transcribing enhancers. In addition, Pol II mediated transcription in wheat is poorly understood. Therefore, we believe combination of pNET-seq and GRO-seq may be the best solution to identify intergenic transcription at current stage.*

*Figure S4. Comparison of GRO-seq and pNET-seq in nascent RNA detection. a, Correlation of read density of transcription clusters from genic and intergenic regions detected by GRO-seq and pNET-seq. b, Venn of intergenic transcription clusters detected by GRO-seq and pNET-seq.*

*As for the antibody issue. We performed the western blot using total protein extracts from 10-day old wheat seedlings. To verify whether the antibody (Ab817) used for pNET-seq can recognize the phosphorylated CTD isoforms of wheat RNA polymerase II, we also included the kinase inhibitor flavopiridol (FLP) treatment, which has been shown to block phosphorylation of Pol II CTD in Arabidopsis. Both phosphorylated and unphosphorylated Pol II were detected by Ab817, while the phosphorylated form was decreased by FLP treatment together with the phosphorylated Pol II forms detected by CMA602 (S2P specific) and CMA603 (S5P specific). Therefore, we believe that at least some of S2P Pol II can be detected by the antibody (Ab817) here.*

*Figure S19. Antibody (ab817) used for pNET-seq can recognize the both unphosphorylated and phosphorylated CTD isoforms of wheat RNA polymerase II. Immunoblots of Pol II CTD phosphorylation in control and seedlings after a 2-hour treatment with flavopiridol (FLA). FLA has been shown to inhibit phosphorylation of Pol II CTD heptamer repeats at Ser2 and Ser5 in Arabidopsis. After FLP treatment, the signal intensity by CMA602 (S2P specific) and CMA603 (S5P specific) decreased, suggesting that FLP block Pol II phosphorylation in wheat seedling as well. Two bands were labelled by ab817, while the upper band was decreased by FLA treatment. Taken together, ab817 can detect unphosphorylated and phosphorylated CTD isoforms of wheat Pol II. Coomassie Brilliant Blue staining (CBB staining) of rubisco large subunit was used as a loading control. IIo and IIa indicate phosphorylated and unphosphorylated Pol II.*

*We added the description in the methods section as “Ab817 was shown to recognize both unphosphorylated and phosphorylated wheat RNA Polymerase II by immunoblotting with total protein from control and flavopiridol treat seedling tissue (Additional file 1: Figure S18)”. Lines 257-260.*

5. Line 176 to 182, the author looked the sub-genome divergent gene expression in relation to sub-genome-biased enhancer transcription. The explanation of the data is simply too brief for readers to understand it. I found the data in Figure 2i is not strong enough to draw a big conclusion (for example, in abstract "sub-genome-biased enhancer transcription is a potential driving force for sub-genome-biased gene expression"), as the majority of enhancers in these plot are not transcribed (gray dot), so a predominant contribution of enhancer transcription in sub-genome-divergent gene expression is not well supported by data. More analysis should be done to clarify this issue.

*Rely: We are sorry that we didn’t present the statistics and conclusions clearly. To be clear, in Figure 2i and 2j, each dot represents a gene pair in which one homeolog is associated with at least one transcribed enhancer in the same chromosome, while the other homeolog is not. The X-axis and Y-axis coordinates represent the expression levels of the two homeolog genes respectively. Red dots represent gene pairs in which a homeolog gene associated with transcribed enhancer(s) was significantly higher expressed than the other one not associated with transcribed enhancer; while the grey dots represent a homeolog gene associated with transcribed enhancer(s) was equally or lower expressed than the other one. The analysis indicates that homeolog genes associated with transcribed enhancers are more likely expressed higher than the ones not associated with transcribed enhancers (red dots), at an odds ratio >1; while the rest scenario (gray dots) are less likely, at an odds ratio < 1. To be cautious we changed the claim “sub-genome-biased enhancer transcription is a potential driving force for sub-genome-biased gene expression” to “sub-genome-biased enhancer transcription could be one of the reasons for sub-genome-biased gene expression”. Lines 26-27.*

*We added description to the legend of Fig2i as “Subgenome biased transcribed enhancers are enriched with unbalanced expressed homoeologs between each two subgenome pairs (from pNET-seq data). Each dot represents a gene pair in which one homeolog is associated with at least one transcribed enhancer in the same chromosome, while the other homeolog is not. The X-axis and Y-axis coordinates represent the expression levels of the two homeolog genes respectively. Red dots represent gene pairs in which a homeolog gene associated with transcribed enhancer(s) was significantly higher expressed than the other one not associated with transcribed enhancer; while the grey dots represent a homeolog gene associated with transcribed enhancer(s) was equally or lower expressed than the other one. “A w/ transcribed enhancer, B w/o transcribed enhancer” means homeolog A is associated with transcribed enhancer(s), homeolog B is not. The odds ratio (OR) is a ratio of two sets of odds. See detailed analyses and interpretation in Additional file 1: Figure S15. j Merge all homoeolog pairs in i showed that the dominantly expressed homoeologs essentially associated with transcribed enhancers”. Lines 573-585.*

*We added a diagram and corresponding legend (FigureS15) to more clearly explain the analysis process and interpretation of results. To the text “using the odds ratio (OR) to measure how strongly “One homeolog gene being expressed higher than the other gene” is associated with “One is associated with transcribed enhancer(s) but the other is not (Additional file 1: Figure S15)” was added. Lines 198-201.*

*Figure S15. Analysis scheme of correlation between sub-genome-divergent gene expression and sub-genome-biased enhancer transcription.*

*In most cases, there is a copy of a homeolog gene for each of the three subgenome homeologous sites. These three genes are referred to as a triad. For the sake of correlation analysis, we focused on 67,108 pairs of homoeolog genes that have 1:1 correspondence between any two of the three subgenomes.*

*Taking the homeolog genes from A and B subgenomes as example, gene pairs can be divided into four scenario: I, homeolog A is associated with transcribed enhancer(s), homeolog B is not; II, homeolog B is associated with transcribed enhancer(s), homeolog A is not; III, both of homeolog A and B are associated with transcribed enhancer(s); IV, neither of homeolog A and B is associated with a transcribed enhancer. We named scenario I as “A W, B W/O” and scenario II-IV as “other”. (In the case that homeolog B is associated with at least one transcribed enhancer, while homeolog A is not, the same statistics are performed. Since A and B are symmetric, only the former case is discussed below.) Based on the expression level, all the gene pairs can be divided into: “A > B” and “A <= B”. Thus, all the gene pairs are divided into four quadrants, z1 (red dots, “A W, B W/O” ∩ “A > B”), z2 (grey dots, “A W, B W/O” ∩ “A <= B”), x, and y.*

*We use the odds ratio (OR) to measure how strongly “A being expressed higher than B” is associated with “A is associated with transcribed enhancer(s) but B is not”. The odds ratio is a ratio of two sets of odds:*

*The formula for calculating the odds ratio (OR) are listed aside.*

*OR > 1 (red dots, odds1/odds2) means that “A is associated with transcribed enhancer(s) but B is not” increase the likelihood of “homeolog A being expressed higher than homeolog B”; while OR < 1 (grey dots, odds2/odds1) means that “A is associated with transcribed enhancer(s) but B is not” decrease the likelihood of “homeolog A being expressed no higher than homeolog B”.*

*Taken together, it is suggested that biased expression of enhancers associated with homeolog A and B could be a reason for asymmetric expression of homeolog A and B themselves.*

Minor point:

1. The colors in Figure 1a is too similar between each features. More contrasting colors should be used.

*Reply: Thanks for the suggestion. We have made the Figure1a using more contrasting colors.*

*Fig 1a. The fractions of GRO-seq and pNET-seq reads aligned to different genomics regions. TSS1k indicated the 1 kb upstream region of the gene transcription start site. TES1k indicated the 1 kb downstream region of the transcription end site/polyadenylation site.*

Reviewer #2:

Combining nascent RNA sequencing and epigenome profiling, the authors identified a quarter of "epigeneticly defined enhancers" showing active transcription in bread wheat genome. They also found that the transcriptional enhancers were associated with active epigenetic makers and higher gene expression levels, indicating that enhancer transcription can potentially be used as a hallmark in regulation of plant gene expression. The prediction of regulatory elements based on enhancer transcription is interesting as it may be complementary to the widely used ATAC-seq, DNase-seq, and MH-seq methods.

*Reply: We thank the reviewer#2 for the constructive feedback.*

Major comments:

This would be the first paper demonstrating transcription of active enhancers in plants. Thus, a conclusive wet lab validation of the predicted enhancers should be absolutely required, especially for a high profile journal like GB. I do not agree that a transient and heterologous transcription system is acceptable for such a validation. Wheat and tobacco belong to monocot and dicot plant species, respectively. It is not acceptable to examine the function of a wheat enhancer in such a distantly related plant species for a concept-establishing manuscript. The authors need to use transgenic assays in wheat or CRISPR/cas based deletion assays to validate a subset of wheat enhancers predicted based on transcription.

*Reply: Thanks for the critical comments. We do agree that the heterologous transcription system such as tobacco leaf may introduce artifacts. Therefore, we tried to test a subset of candidate enhancers in a wheat protoplast system. Of 39 candidate enhancers already tested in tobacco, we successfully tested 36 with the dual luciferase reporter system and observed 21 positive and 15 negative outcomes in activating reporter expression using 2-fold increase of relative luciferase activity as cut-off.*

*Moreover, the positive candidate enhancers validated in the wheat protoplasts had higher transcription activity than the negative ones in wheat seedlings. The transcribed candidate enhancers are more active in wheat protoplasts.*

*Figure S14. Test activity of enhancer candidates in wheat protoplasts. a, Examples of GFP expression driven by mini35S or mini35S+enhancer in wheat protoplasts. b, pNET-seq (left) and GRO-seq (right) signal density in positive (P) or negative (N) candidate enhancers determined in wheat protoplasts.*

*Fig 1e. The activity (relative intensity) determined in the wheat protoplast reporter assay of enhancer candidates with or without transcription.*

*Furthermore, enhancer activity determined in wheat protoplasts are well correlated with nascent RNA intensity, followed by chromatin openness, H3K9ac, and H3K27ac etc. Therefore, similar conclusions were drawn from the data of wheat protoplasts, and we decided to replace the tobacco results in the original manuscript with wheat protoplast results. We plan to test the activity of positive enhancers through gene editing and transgenic wheat in the future.*

*Fig 2f. Correlation between the relative intensity and the nascent RNA sequencing, DSH, and ChIP-seq read density in transcribed predicted enhancer regions.*

*We also modified the description in the text as “We then asked whether enhancer transcription level was related to enhancer activity. We leveraged the “enhancer activity” of 36 putative intergenic enhancers, which was determined using a luciferase reporter assay in wheat protoplasts (Fig. 2e, Additional file 1: Figure S1, S14a). The transcribed candidate enhancers are more active in wheat protoplasts (Fig. 2e). Furthermore, the positive candidate enhancers validated in the wheat protoplasts had higher transcription activity in wheat seedlings (Additional file 1: Figure S14b). Pearson correlation coefficients were calculated between the relative luminescence intensities (enhancer activity) and the read densities of nascent RNA (pNET-seq and GRO-seq) and different chromatin marks. Notably, enhancer activity was the most positively correlated to enhancer transcription (GRO-seq and pNETseq, r = 0.55), followed by chromatin openness (DHS, r = 0.43) and active histone modification (H3K9ac, r =0.17; and H3K27ac, r =0.16), which indicated eRNA is more robust in predicting enhancer activity as compared to canonical chromatin features (Fig. 2f)”. Lines 171-183.*

Minor comments:

Library constructions for GRO-seq and pNET-seq are tricky especially in plants. The manuscript lacks evidence to prove the data quality of GRO-seq and pNET-seq. (a) Does most of the enhancer transcription overlap between the replicates of GRO-seq or pNET-seq data? (b) Does most of the enhancer transcription overlap between GRO-seq and pNET-seq data? (c) Since the nascent RNA transcription was detected, why the numbers of reads mapped to exon and intron regions were so different in Figure 1a? (d) How the "TSS 1k" was defined in Figure 1a? Why the proportion of the reads mapped to "TSS 1k" was only ~5%, considering "transcriptional pausing was observed in close proximity to active transcription start sites".

*Reply: Thank you for pointing out these confusing points. Yes, we do agree that GRO-seq and pNET-seq are tricky experiments. That is why we performed multiple independent experiments for pNET-seq and GRO-seq. In this version, we re-did the GRO-seq and included more data (also see the response to reviewer#1 point4).*

*a) The overall correlation coefficient between GRO-seq and pNET-seq is good (0.57 and 0.60 at genic and intergenic regions, respectively) (FigureS4), especially for a genome as complex and large as wheat. The correlation coefficient of the newly included GRO-seq and pNET-seq replicates were above 0.96 (also see the response to reviewer#1 point4, FigureS4).*

*b) A large portion of transcribed enhancers (6,342) were detected by both methods. And some of transcribed enhancers were detected by either method (5,142 by GROseq and 6,345 by pNET-seq).*

*c) Most of the reads are mapped to the first exon in 5’end of genes, probably due to the proximal promoter pausing (FigureS2b). If we classify exons to exon_1 and exon_other, the difference between exon_other and intron is very small. And when included more GRO-seq data, the distribution is even between GRO-seq and pNETseq (Fig1a).*

*Figure for response. The raw read count (left) and normalized read count (right) aligned to different genomics regions. The genome length is used to normalize.*

*d) TSS1k is 1kb upstream region of the gene transcription start site annotated according to IWGSC1.1. Because a larger proportion of reads are mapped to the first exon which is downstream to the TSS, the proportion of the reads mapped to “TSS 1k” is small. To avoid confusion, we have added explanations to the Figure1 legend “TSS1k indicated the 1 kb upstream region of the gene transcription start site. TES1k indicated the 1 kb downstream region of the transcription end site/polyadenylation site”. Lines 543-544.*

Are the proportions of intergenic enhancers producing transcripts in state 5-7 significantly higher than other state groups? (page5: line 108-110)

*Reply: Yes. We plotted the transcribed fractions of state 1-15. State 5-7 showed higher transcription than the other states by pNET-seq. State 5 showed higher transcription than other states and state 6-7 showed comparable transcription to state 2-4 (genic region) by GRO-seq.*

*Figure S9. The active transcribed (orange) and non-transcribed (green) fractions in states 1-15 detected by GRO-seq (left panel) and pNET-seq (right panel).*

*We included these data in the FigureS9 and added description to the text “the proportions of intergenic enhancer-like elements being transcribed in states 5-7 were significantly higher than those in other states”. Lines 120-122.*

The authors appear to fully ignore the recent plant enhancer research based on open chromatin-mediated prediction. The authors need to review these recent and relevant research work.

*Reply: We are sorry that we didn’t quote these important literatures. We have added the literatures. Line 39.*

*[1-4]*

*1. Zhu B, Zhang W, Zhang T, Liu B, Jiang J: Genome-Wide Prediction and Validation of Intergenic Enhancers in Arabidopsis Using Open Chromatin Signatures. The Plant Cell 2015, 27:2415-2426.*

*2. Oka R, Zicola J, Weber B, Anderson SN, Hodgman C, Gent JI, Wesselink J-J, Springer NM, Hoefsloot HCJ, Turck F, Stam M: Genome-wide mapping of transcriptional enhancer candidates using DNA and chromatin features in maize. Genome Biology 2017, 18:137.*

*3. Meng F, Zhao H, Zhu B, Zhang T, Yang M, Li Y, Han Y, Jiang J: Genomic editing of intronic enhancers unveils their role in fine-tuning tissue-specific gene expression in Arabidopsis thaliana. Plant Cell 2021, 33:1997-2014.*

*4. Zhang W, Wu Y, Schnable JC, Zeng Z, Freeling M, Crawford GE, Jiang J: High-resolution mapping of open chromatin in the rice genome. Genome research 2012, 22:151-162.*

**Second round of review**

**Reviewer 1**

In the revised version of the manuscript. The authors performed additional experiments and analyses to address my questions correctly. In the meantime, a few minor points should be addressed further. In brief, please notify two points. First, current data does not support transcribed enhancers in wheat is very different in terms of histone modifications compared with the generic region; this should be clearly stated somewhere in the abstract or results. Second, the enhancer activity validation part needs to be written more clearly with all relevant information. Below, I detail my comments.

1. The Last sentence of Abstract, to be specific, this reviewer found the study highlights the enhancer transcription in wheat but not in plants which is a too broad definition.

2. Line 55-56, pNET-seq signal does not always equal to active transcription, so please be precise here.

3. Line 61-63, it is unclear why the aligned reads derived from exon in pNET-seq/GRP-seq is expected; please explain briefly. I found it's interesting that the other highest proportion is from intergenic regions. I am a little concerned here that most intergenic pNET-seq signals are irrelevant of the enhancer. For example, Pol II 3'end peak downstream of polyA site, which could be counted as intergenic signals. Could the author clarify this issue? Also, what's the situation in Arabidopsis? In other words, is this phenomenon wheat specific? This kind of comparison will benefit the readers for sure. And the data is available as the same group publishes it.

4. Fig S2B, TES should be polyadenylation site instead? Otherwise, it is unclear how transcription termination/end site can be defined.

5. Line 94-95, the intergenic transcribed region has little or no association with H3K4me1, H3K9me2 and H3K27me3. What's the implication of this result? H3K9me2 is a hallmark pericentromeric region and transposable element, while H3K27me3 is a repressive mark on euchromatin. I don't think these two marks are associated with active/transcribed regions in either intergenic or genic regions. If the author shows the results of un-transcribed genes or intergenic regions, these two marks could be associated with those regions much stronger. To avoid confusion, I suggest dropping out the H3K9me2 and H3K27me3 results. H3K4me1 result is somewhat more interesting, and please explain it in a more detailed way with caution.

6. Line 169-170, enhancers in plant should be in wheat here.

7. Figure S12 and S13, no figure legend is provided. Please note the SI figure should be treated to the same standard as the main Figure.

8. Line 171 and 183, the updated enhancer validation. I found the information provided is not sufficient to judge the results. Line 173-174, please briefly introduce the assay, what's the design and controls. The SI figure legend is too brief, the same for the method section. Line 174-175, "more active in wheat protoplast", what was compared with is missing here.

**Reviewer 2**

My major concern of the original manuscript was the validation of the predicted enhancers based on a transient and heterologous transcription system. The authors used a protoplast-based transient system for enhancer validation in this revised manuscript. This is clearly not an ideal method for enhancer validation, especially for a concept-establishing manuscript. Nevertheless, I appreciate the authors' effort and am willing to accept this approach. However, the current manuscript lacks details of the protoplast-based experiments: what are the negative controls? How these controls were selected? How many were used? Each construct was tested by a single or multiple experiments? A supplemental table with numerical data of each construct should be provided.

**Authors Response**

**Point-by-point responses to the reviewers’ comments:**

**Reviewer #1:**

In the revised version of the manuscript. The authors performed additional experiments and analyses to address my questions correctly. In the meantime, a few minor points should be addressed further. In brief, please notify two points. First, current data does not support transcribed enhancers in wheat is very different in terms of histone modifications compared with the generic region; this should be clearly stated somewhere in the abstract or results. Second, the enhancer activity validation part needs to be written more clearly with all relevant information. Below, I detail my comments.

*Reply: We appreciated the constructive suggestions and made the changes accordingly.*

*For the difference between transcribed enhancers and genes, we added sentences into the conclusion section “The overall chromatin signature of transcribed enhancers is quite similar to that of actively transcribed genes, marked with open chromatin, H3K9ac, H3K4me3, H3K36me3 and H3K27ac. However, bidirectional transcription was often observed at enhancers rather than at genic regions.” Line 238-241.*

*In addition, we have provided more information of validation experiments in wheat protoplasts. See below.*

1. The Last sentence of Abstract, to be specific, this reviewer found the study highlights the enhancer transcription in wheat but not in plants which is a too broad definition.

*Reply: Thanks for the suggestion. We have made the change. Also, in the conclusion we deleted “plant kingdom”, Line 237.*

2. Line 55-56, pNET-seq signal does not always equal to active transcription, so please be precise here.

*Reply: Thank you for your comments. Yes, pNET-seq signal can indicate either ongoing or stalled Pol II in certain DNA regions that are being or have been transcribed. So, we have deleted the word “actively”. Line 55.*

3. Line 61-63, it is unclear why the aligned reads derived from exon in pNET-seq/GRP-seq is expected; please explain briefly. I found it's interesting that the other highest proportion is from intergenic regions. I am a little concerned here that most intergenic pNET-seq signals are irrelevant of the enhancer. For example, Pol II 3'end peak downstream of polyA site, which could be counted as intergenic signals. Could the author clarify this issue? Also, what's the situation in Arabidopsis? In other words, is this phenomenon wheat specific? This kind of comparison will benefit the readers for sure. And the data is available as the same group publishes it.

*Reply: Thanks for pointing out the confusing claims. We have added the explanation “In the genome, genic regions are considered to be transcribed. Pol II-mediated transcription is slower in exons than in introns to facilitate splicing recognition. Therefore, it is likely that more nascent RNAs are detected in exons than in introns [25].” Line 61-64.*

*The intergenic regions we defined here did not include 1 kb upstream and downstream of gene regions, thus excluding the Pol II 3’ end peak downstream of the polyA site. The proportion of Arabidopsis GRO-seq clusters from the intergenic regions is 1%. According to our definition, intergenic regions account for 18% of the Arabidopsis genome, and nascent RNAs were detected in 19% of intergenic regions by GRO-seq. We add “In contrast, the Arabidopsis genome is less than 1% of the wheat genome, and only 18% is intergenic. Transcription was detected in 19% of Arabidopsis intergenic regions, accounting for 1% of nascent RNAs detected by GRO-seq (Additional file 1: Figure S2a)”. Line 66-69.*

*Figure S2a GRO-seq reads aligned to different Arabidopsis genomic regions. TSS1K indicates the 1 kb upstream region of the gene transcription start site. TES1K indicates the 1 kb downstream region of the gene transcription end site/polyadenylation site.*

4. Fig S2B, TES should be polyadenylation site instead? Otherwise, it is unclear how transcription termination/end site can be defined.

*Reply: TES is the 3’end of gene annotated by IWGSC Ref1.1. It was defined using RNA-seq, ISOseq and flcDNA data. To clarify the definition, we changed the TES into PAS/TES.*

5. Line 94-95, the intergenic transcribed region has little or no association with H3K4me1, H3K9me2 and H3K27me3. What's the implication of this result? H3K9me2 is a hallmark pericentromeric region and transposable element, while H3K27me3 is a repressive mark on euchromatin. I don't think these two marks are associated with active/transcribed regions in either intergenic or genic regions. If the author shows the results of un-transcribed genes or intergenic regions, these two marks could be associated with those regions much stronger. To avoid confusion, I suggest dropping out the H3K9me2 and H3K27me3 results. H3K4me1 result is somewhat more interesting, and please explain it in a more detailed way with caution.

*Reply: Yes, H3K9me2 is a major marker of heterochromatin. However, under the balance of IBM1 (H3K9 demethylase) and SUVH4 (H3K9 methylase), certain gene body regions of Arabidopsis may be marked by H3K9me2. While the regions marked by both H3K4me3 and H3K27me3 are referred to as the poised state. We noticed that even within the genic region the association with H3K9me2 or H3K27me3 was somewhat weak. We agreed to remove the H3K9me2 and H3K27me3 results to avoid potential confusion. Intergenic regions have weaker levels of H3K4me1 compared to genic regions, suggesting that H3K4me1 is not a sensitive marker for predicting transcriptional enhancers. It is indeed interesting that H3K4me1 was marginally correlated with intergenic nascent RNA levels despite its low abundance. Thus, we added “Consistent with previous plant epigenome studies [1, 30], more H3K4me1 was detected in gene bodies than in intergenic regions (Fig. 1d; Additional file 1: Figure S5). However, there was a weak correlation between nascent RNA abundance and H3K4me1 levels in intergenic regions, whereas there was no clear relationship between H3K4me1 and nascent RNA levels in genes (Fig. 1f; Additional file 1: Figure S6, S7).” Line 109-114.*

6. Line 169-170, enhancers in plant should be in wheat here.

*Reply: Thanks for the suggestion. We have corrected it.*

7. Figure S12 and S13, no figure legend is provided. Please note the SI figure should be treated to the same standard as the main Figure.

*Reply: Thanks for the suggestion. We have added necessary information into the figure legend. “Figure S12. The correlation between enhancer transcription or chromatin states and target gene expression. a-g, Boxplots showing transcript levels of target genes(by RNAseq) associated with enhancers with different eRNA levels (a-b), histone modifications (c-f), and chromatin accessibility (g) levels. The target gene expression positively correlates with the associated enhancer transcription level by GRO-seq (a) and pNETseq (b), and the H3K9ac signals (c), but not with other histone modification (d-f) or chromatin accessibility (g).”*

*“Figure S13. The expression levels of target genes are independent of the distance, position, and orientation of associated transcribed enhances. a-f, Boxplots showing transcript levels of target genes associated with transcribed enhancers with different distances (a, d), positions (b, e), and orientations (c, f). The target gene expression does not correlate with the distance, position, and orientation of associated transcribed enhancers identified by GRO-seq (a-c) or pNET-seq (d-f).”*

8. Line 171 and 183, the updated enhancer validation. I found the information provided is not sufficient to judge the results. Line 173-174, please briefly introduce the assay, what's the design and controls. The SI figure legend is too brief, the same for the method section. Line 174-175, "more active in wheat protoplast", what was compared with is missing here.

*Reply: Thanks for the suggestion.*

*To provide details for enhancer validation, we have added a FigureS14 and rewrote the Methods section. Please also refer to the response to reviewer #2.*

*We have added a brief introduction and clarified the confusion in the text “We then asked whether the enhancer transcription level was related to enhancer activity. We tested the “enhancer activity” of 36 putative intergenic enhancers with or without eRNA, which was determined using gfp or dual luciferases as reporters in wheat protoplasts (see Methods; Additional file 1: Figure S14, S1). The putative enhancer region was fused with a 35S minimal promoter (mini35S pro) controlling the transcription of reporter genes, and the “enhancer activity” was defined as its effect on the reporter gene expression in comparison to the negative and blank controls, measured qualitatively by fluorescence of GFP or quantitatively by luciferase activity. The transcribed candidate enhancers were more active than untranscribed ones in wheat protoplasts (Fig. 2e; Additional file 1: Figure S15a).” Line180-189.*

*The FigureS15 legend was modified as “Figure S15. Test enhancer activity in wheat protoplasts.*

*a, Wheat protoplasts transfected with the blank control vector (mini35Spro:gfp, left) or vectors with enhancer candidates (enhancer-mini35S:gfp, middle and right). The GFP signal in the control experiment was weak and confined to the nucleus, whereas those with enhancer-mini35s:gfp had a bright GFP signal distributed throughout the cell.*

*b, pNET-seq (left) and GRO-seq (right) signals are significantly higher in positive (P) than negative (N) candidate enhancers determined in wheat protoplasts (t.test, p=0.049 for pNET-seq and p=0.011 for GRO-seq).”*

**Reviewer 2**

My major concern of the original manuscript was the validation of the predicted enhancers based on a transient and heterologous transcription system. The authors used a protoplast-based transient system for enhancer validation in this revised manuscript. This is clearly not an ideal method for enhancer validation, especially for a concept-establishing manuscript. Nevertheless, I appreciate the authors' effort and am willing to accept this approach. However, the current manuscript lacks details of the protoplast-based experiments: what are the negative controls? How these controls were selected? How many were used? Each construct was tested by a single or multiple experiments? A supplemental table with numerical data of each construct should be provided.

*Reply: Thank you for recognizing our efforts. Due to the word limit of short report, we are sorry that we didn't provide enough experimental details.*

*In the wheat protoplast system, gfp and luciferase genes were adopted as qualitative and quantitative reporters respectively. The mini 35S promoter driven gfp or firefly luciferase was used as a blank control, indicating a basal level of transcription. An intergenic region, where there is no DHS, pNET-seq, and GRO-seq signal, fused with the mini 35S promoter was chosen as a negative control, indicating no or low enhancer activity. For each construct/putative enhancer, three biological replicates were performed. The Renilla luciferase driven by a UBQ10 promoter was used to normalize the transfection differences among multiple experiments. Information such as enhancer locations, eRNA levels, and the relative signal densities from the reporter assay (relative intensity/enhancer activity of each replicate and the average value of the triplicates) are presented separately in the supplemental Table S6. Also see the response to reviewer#1 point8.*

*We have added a Figure S14 to describe the constructs and calculation of relative intensity/enhancer activity in detail. The Methods section was rewritten as “Verification of enhancer activity by a luciferase reporter assay in wheat protoplasts Sequences of the predicted intergenic enhancers were amplified from Chinese Spring genomic DNA (primers are listed in Additional file 2: Table S5) and fused with a minimal 35S promoter, which drives the transcription of a Fluc (firefly luciferase) or gfp (green fluorescent protein) gene [1] (Additional file 1: Figure S14). Wheat protoplast preparation and transformation were performed according to a previously reported protocol [54]. Briefly, wheat leaves were cut into strips with a razor blade and then incubated with cellulase and macerozyme to release protoplasts. A total of 15 μg plasmids were transformed into 1×105 protoplasts using the PEG-mediated transfection method. After 24 h of incubation under dark conditions at room temperature, protoplasts were collected for visualization of fluorescence or luciferase assays. The protoplasts transformed with gfp reporter constructs were observed with a confocal microscope (Nikon C2 plus) (Additional file 1: Figure S15). For the dual luciferase reporter system, Rluc (Renilla luciferase) driven by a UBQ10 promoter was used to monitor transfection efficiency in the same vector (Additional file 1: Figure S14). The luciferase assay was completed using the Dual-Luciferase Reporter Assay System (Promega) according to the manufacturer’s instructions on a BioTek Synergy 2 microplate reader. The relative expression level of each construct was defined as the ratio of Fluc to Rluc activity. To calculate the enhancer activity (relative intensity), the relative expression level of each enhancer candidate construct was normalized to that of the blank control, in which Fluc was only driven by the minimal 35S promoter. An intergenic region where there were no DHS, pNET-seq and GROseq signals was used as a negative control. The average of three independent biological replicates was recorded for each construct. The enhancer activity (relative intensity) of the negative control was 1.6; thus, we set 2.0 as the cutoff of the positive enhancer. The activity for each enhancer candidate is listed in Additional file 2: Table S6. For correlation coefficient calculation between enhancer activity and eRNA/histone modification/DHS levels, extreme out group points were omitted.” Line369-395.*
